# Self-organized BMP signaling dynamics underlie the development and evolution of digit segmentation patterns in birds and mammals

**DOI:** 10.1073/pnas.2304470121

**Published:** 2024-01-04

**Authors:** Emmanuelle Grall, Christian Feregrino, Sabrina Fischer, Aline De Courten, Fabio Sacher, Tom W. Hiscock, Patrick Tschopp

**Affiliations:** ^a^Zoology, Department of Environmental Sciences, University of Basel, Basel 4051, Switzerland; ^b^Institute of Medical Sciences, University of Aberdeen, Aberdeen AB25 2ZD, Scotland, United Kingdom

**Keywords:** digit development, Turing mechanisms, cell fate decisions, synovial joints, mathematical modeling

## Abstract

Repeating patterns of synovial joints are a highly conserved feature of articulated digits, with variations in joint number and location resulting in diverse digit morphologies and limb functions across the tetrapod clade. During the development of the amniote limb, joints form iteratively within the growing digit ray, as a population of distal progenitors alternately specifies joint and phalanx cell fates to segment the digit into distinct elements. While numerous molecular pathways have been implicated in this fate choice, it remains unclear how they give rise to a repeating pattern. Here, using single-cell RNA sequencing and spatial gene expression profiling, we investigate the transcriptional dynamics of interphalangeal joint specification in vivo. Combined with mathematical modeling, we predict that interactions within the BMP signaling pathway—between the ligand GDF5, the inhibitor NOGGIN, and the intracellular effector pSMAD—result in a self-organizing Turing system that forms periodic joint patterns. Our model is able to recapitulate the spatiotemporal gene expression dynamics observed in vivo, as well as phenocopy digit malformations caused by BMP pathway perturbations. By contrasting in silico simulations with in vivo morphometrics of two morphologically distinct digits, we show how changes in signaling parameters and growth dynamics can result in variations in the size and number of phalanges. Together, our results reveal a self-organizing mechanism that underpins amniote digit segmentation and its evolvability and, more broadly, illustrate how Turing systems based on a single molecular pathway may generate complex repetitive patterns in a wide variety of organisms.

## Significance

Tetrapod digits are segmented into individual bones, the phalanges, which are connected by synovial joints, with variations to phalanx number and size generating diverse limb morphologies. Given the range of joint numbers within a given digit—from 1 in the human thumb to over 10 in the second digit of certain species of whale—it has been speculated that self-organizing mechanisms may underlie the initiation of these repetitive segmentation patterns. Here, using single-cell molecular profiling, in vivo growth series, and mathematical modeling, we uncover a self-organizing Turing system that determines digit segmentation. These results have implications for our understanding of amniote digit patterning and its evolutionary diversification, as well as for the etiology of human congenital malformations of the hands and feet.

Articulated digits with repeating joints are a hallmark of the distal tetrapod limb and considered an essential early adaptation to locomotion on dry land (1–3). Digits are segmented into individual digit bones, termed phalanges, which are connected to each other by synovial, interphalangeal joints that facilitate relative motion of adjacent bones and, thus, digit flexion. Thanks to this modular architecture, highly distinct digit patterns have evolved to enable locomotory behaviors as diverse as walking, swimming, flying, or the execution of fine motor skills. These patterns vary both in terms of sizes of their phalangeal bones, as well as the number of segments and—accordingly—interphalangeal joints. For example, the human thumb consists of two phalanges, connected by a single joint, while the rest of the digits are made up of three individual digit bones. In chicken feet, the number of phalanges per digit ranges from two to five, in bat wings from one to three, with over ten phalanges being present in the second digit of certain species of whale (4). Variations in phalangeal numbers within a species are considered a manifestation of distinct digit identities along the anteroposterior axis of the distal limb, the so-called autopod (4, 5).

**Fig. 1.**
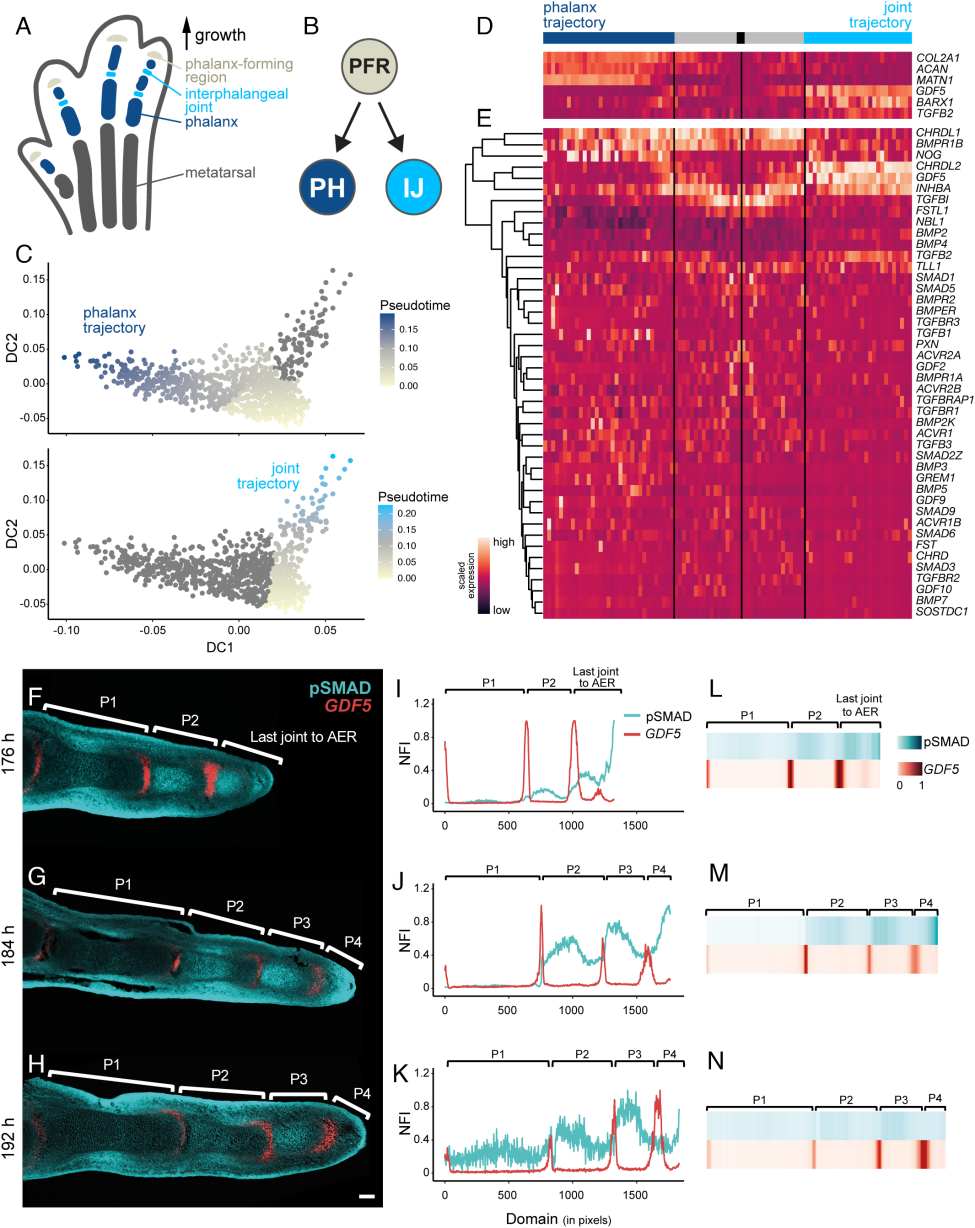
TGF-beta signaling dynamics during the phalanx/joint cell fate decision. (*A*–*E*) Pseudotime single-cell RNA-seq analyses of the phalanx/joint-progenitor cell fate decision in the chicken hindlimb. (*A*) Schematic of the different elements in a developing digit at stage HH29. (*B*) A bifurcating cell fate decision splits the PFR cell population into either phalanx (PH) or interphalangeal joint (IJ) progenitor cells. (*C*) Pseudotime progression along the phalanx and the joint trajectories. (*D* and *E*) Heatmap of differentially expressed genes along the phalanx progenitor (dark blue) or joint progenitor (turquoise) trajectories. The black box corresponds to the starting point, gray zones to the part shared by the two trajectories. Scaled gene expression from low (purple) to high (orange). (*E*) Hierarchically clustered pseudotime heatmap of all detected genes of the TGF-beta superfamily. (*F*–*N*) Spatiotemporal in vivo data for *GDF5* gene expression and BMP signaling activity in developing digits. (*F*–*H*) Fluorescent RNA in situ hybridization for *GDF5* combined with immunohistochemistry for pSMAD on longitudinal sections of digit III at 176 h, 184 h, and 192 h of development. (Scale bar, 100 μm.) (*I*–*K*) Plots of normalized fluorescence intensities (NFI) for pSMAD (cyan) and *GDF5* (red) along the proximal–distal axis of digit III. 1 pixel = 1.243 μm. (*L*–*N*) Heat map visualizations of NFI for pSMAD (cyan) and *GDF5* (red) of digit III.

The foundation for this diversity in digit morphologies is laid down early during embryonic patterning. Once the three major proximal-distal segments of the tetrapod limb have been defined—the stylopod (upper arm/leg), zeugopod (forearm/lower leg) and autopod (hand/foot)—digit outgrowth in amniotes is initiated at the distal margin of the autopod, through the specification of a progenitor population at the distal digit tip known as the phalanx forming region (PFR) (6–8). Molecular and mechanical cues converge to shape this organizing center, with fibroblast growth factors (FGFs) from the overlaying apical ectodermal ridge (AER) defining a distal domain of growth competency (9–11). Cell rearrangements at the PFR result in rod-shaped and elongating digital rays, as progenitors become incorporated distally (10) (Fig. 1*A*). Once skeletogenic digit progenitors leave the signaling environment of the PFR niche, they differentiate into either chondrocytes, the phalanx progenitors, or interzone cells, which mark the site of future joints (12, 13). By alternately differentiating into chondrocyte or interzone cell fates, the digit thus forms a repeating sequence of phalanges and joints. Accordingly, while the final number of phalanges correlates with the overall duration of digit ray elongation (11, 14), the initial size of skeletal elements is defined by the periodicity with which either of the two cell types is sequentially specified (Fig. 1 *A* and *B*).

This alternating pattern of cell fates is first observable as periodic stripes of gene expression of joint cell fate markers along the proximal-distal axis of the digit. Given the aforementioned diversity in eventual joint numbers, this suggests that the locations of these early stripes of marker gene expression are not set individually, but rather are the result of an inherently iterative developmental mechanism that places joints at defined spatial intervals along the digit. Indeed, previous studies have proposed that self-organizing, potentially Turing-like mechanisms might be responsible for the development of these patterns (13, 15–20). In a Turing-like scenario, a network of interacting and diffusing biochemical signals forms a reaction–diffusion system that self-organizes into a periodic pattern (21). The core underlying principle here is the presence of a long-range negative feedback loop in the system, typically achieved by a rapidly diffusing inhibitor molecule, whose diffusive range sets the characteristic spacing of the pattern. As such, Turing mechanisms allow repetitive structures to form at regular spatial intervals, akin to how joints repeatedly form along the length of the digit. Several experimental observations implicate a Turing-like system in phalanx-joint patterning. First, insertion of a foil barrier into the developing digit perturbs phalangeal proportions, suggesting that molecular diffusion plays an essential role in patterning (22). Second, experimentally induced ectopic joints can repress the formation of endogenous joints, suggestive of Turing-like long-range inhibition (17). Third, certain mouse mutants display aberrant joint patterns predicted to be highly specific to Turing mechanisms (18, 23). However, while these data suggest that digit patterning might be Turing-like, the specific molecular network responsible for patterning remains poorly defined, and it is still unclear how it could generate a self-organizing reaction–diffusion system.

Genetic studies in humans and mice, as well as experimental embryology in avian models, have identified many genes and molecular pathways involved in digit development (e.g., WNT, IHH, FGF) (11, 15, 17, 24, 25). In particular, the bone morphogenetic protein (BMP) pathway, comprising members of the transforming growth factor-beta (TGF-beta) superfamily, appears to play an essential role during phalanx-joint specification. One of the earliest known markers of the joint interzone is the BMP ligand *Growth Differentiation Factor 5* (*GDF5*) (26, 27). GDF5 activates BMP-signaling by binding to its preferred receptor, BMPR1B, which is expressed within the developing digit ray (7, 28). Moreover, high levels of pSMAD1/5/8, the nuclear effectors of active BMP signaling, and pSMAD2/3, downstream of *Activin* signaling, mark the PFR progenitor population and are essential for digit outgrowth (6, 7, 10). Differences in pSMAD1/5/8 levels at the PFRs of digits in the same autopod mirror distinct digit identities and precede the emergence of digit-specific morphologies (29). Finally, congenital defects in human digit patterning are frequently associated with mutations in members of the BMP pathway, including *GDF5, BMPR1B,* and *NOGGIN* (*NOG*), an extracellular BMP inhibitor known to bind GDF5 (30). Based on these observations, it has been hypothesized that regulatory interactions among diffusible members of the BMP pathway may play a critical role in generating the periodic phalanx-joint patterns characteristic of tetrapod digits (13, 15–17, 19).

Here, we combine single-cell transcriptomic data of the PFR and its descendant cell populations—the phalanx and joint progenitors—with quantitative in situ measurements to describe the spatiotemporal dynamics of BMP activity during digit segmentation in vivo. Building on the observed dynamics, we use mathematical modeling to propose a BMP-based Turing system that can recapitulate patterning in silico, as well as phenocopy experimental perturbations affecting its main molecular constituents. Combined with developmental growth and segmentation data from two morphologically distinct digits, we discuss potential cellular and molecular mechanisms underlying the development and evolution of distinct digit patterns in the amniote autopod.

## Results

### Transcriptional Signatures and BMP Signaling Dynamics of the Phalanx-Joint Cell Fate Decision.

To follow the transcriptional dynamics accompanying the specification of PFR progenitors into either phalanx-forming chondrocytes or joint-inducing interzone cells, we explored a single-cell RNA-sequencing (scRNA-seq) dataset of the chicken foot at Hamburger-Hamilton stage 29 (HH29) (31, 32). Due to the growth dynamics of the chicken digit at that stage, sampling the distal autopod of multiple embryos—with slight developmental heterochronies between them—should capture the transcriptional signatures of the PFR, the phalanx- and joint-forming progenitors, as well as the intervening cell states of this cell fate decision process [Fig. 1 *A* and *B*, (31)]. Indeed, based on marker gene expression analyses, we were able to identify three scRNA-seq cell clusters with transcriptional profiles reminiscent of a proliferative distal cell population with an early chondrocyte signature, a maturing chondrocyte population, and cells showing signs of interzone cell fate induction (*SI Appendix*, Fig. S1*A*). Focusing on these three clusters, we selected variable genes to calculate a diffusion map (33), which placed the distal cell population (cluster 15) at the root of a bifurcating cell fate trajectory into either phalanx (cluster 3) or joint progenitors (cluster 17) (*SI Appendix*, Fig. S1*B*). Using Slingshot (34), we calculated pseudotemporal orderings of cells along these two diffusion map branches (*SI Appendix*, Fig. S1*C*), resulting in a “phalanx”- and a “joint”-specific trajectory, respectively (Fig. 1*C*). The top differentially expressed genes along these two trajectories contained many known markers of the respective cell populations, with their expected temporal expression dynamics (Fig. 1*D* and *SI Appendix*, Fig. S1*D*). For example, we were able to detect the expression of PFR markers, such as *INHBA* or *TCF7L2* (7, 8), at the onset of our pseudotime. Genes indicative of a progressive maturation of either phalangeal chondrocytes (e.g., *COL2A1*, *ACAN*, *MATN1*) or joint interzones (e.g., *GDF5*, *BARX1*, *TGFB2*) were up-regulated toward the respective ends of the two trajectories (35). Additionally, we identified novel markers and putative regulators of this bifurcating cell fate decision (*SI Appendix*, Fig. S1*D*). Collectively, using scRNA-seq pseudotime analyses, we reconstructed the transcriptional dynamics at the PFR, documenting the expression dynamics of both known and novel marker genes, as digit progenitors differentiate into either phalanx or joint cell fates.

Given the genetic evidence supporting the importance of the TGF-beta superfamily in digit formation and patterning (30), we next focused on the transcriptional dynamics of all its members whose expression we detected in our HH29 sample. Hierarchical clustering based on expression dynamics across the trajectories revealed high temporal variance in six BMP genes, with three phalanx- and three joint-enriched signatures (Fig. 1*E*). At the onset of our pseudotemporal progression, i.e., corresponding to the PFR and its immediate descendants, we detected *CHRDL1* and *BMPR1B*, whose expression extended into the phalanx branch, as well as *INHBA* (also known as *Activin Beta-A*), which additionally appeared re-activated later in the joint trajectory. After the bifurcation point of the phalanx and joint trajectories, *NOG* was transcribed in the phalanx trajectory and, seemingly, to a lesser extent and with higher variability in the joint trajectory. Also, *GDF5* and *CHRDL2* became expressed specifically in the joint progenitors, with *GDF5* being the only BMP ligand that we detected at appreciable levels in either of the two trajectories. These scRNA-seq expression profiles thus suggested that the majority of BMP activity during the phalanx-joint cell fate decision is driven by GDF5, signaling through its preferred receptor BMPR1B (28, 36).

Accordingly, we next investigated the spatiotemporal profiles of *GDF5* expression and BMP pathway activity in vivo. Using longitudinal tissue sections of chicken foot digit III at different developmental time points, we performed fluorescent in situ hybridization (FISH) for *GDF5* and combined it with fluorescent immunohistochemistry against the phosphorylated versions of SMADs 1, 5, and 9 (referred to as pSMAD thereafter), as a proxy for BMP pathway activity (Fig. 1 *F*–*H*). For both *GDF5* and pSMAD, we quantified normalized fluorescent intensities (NFIs) along the proximal-distal axis of the digit and visualized them using either line plots or heatmaps (Fig. 1 *I*–*N*). This revealed repetitive peaks of restricted *GDF5* expression, corresponding to the forming joints, with broad shoulders of pSMAD activity marking the intervening phalangeal segments. The most distal digit domains, i.e., where full segmentation had yet to occur (“Last joint to AER”), showed particularly dynamic profiles of *GDF5* and pSMAD. Counterintuitively, given its role as a BMP-activating ligand, wherever *GDF5* expression initiated, we observed a drop in pSMAD intensity, rendering the two activity profiles essentially out of phase from one another (Fig. 1 *I* and *L*). This occurs even though the entire distal digit domain expresses BMPR1B (6), i.e., all its cells should be competent to activate BMP signaling via GDF5. We therefore explored whether additional, potentially self-organizing mechanisms could explain the absence of pSMAD at sites of distal *GDF5* transcription and the overall repetitive nature of observed BMP activity.

### Theory Predicts that Spatially Periodic Expression of the Inhibitor *NOG* is Required for Digit Patterning.

Ectopic pSMAD activity is known to down-regulate *GDF5* transcription in a cell-autonomous manner (16). Furthermore, GDF5 protein can diffuse and activate pSMAD at a distance (37). Therefore, GDF5 produced at the joint interzone could diffuse away to activate BMP signaling within the forming phalanx regions, where pSMAD would in turn down-regulate further *GDF5* expression and thereby define antiphasic *GDF5/*pSMAD patterns. Collectively, these interactions delineate a long-range negative feedback loop, wherein diffusible GDF5 inhibits its own expression via activation of pSMAD at a distance. Such long-range inhibition is a hallmark feature of reaction–diffusion-based Turing patterns, with GDF5 fulfilling the requirements of a putative Turing inhibitor. In addition to GDF5—an activator of BMP signaling—we reasoned that the reaction–diffusion system must incorporate an inhibitor of BMP signaling, to restrict pSMAD activity within the initiating joint interzone. Provided the inhibitor is secreted, this would add a second diffusible factor to the proposed network, in addition to GDF5. Importantly, a model based on GDF5 and pSMAD alone—i.e., with only one diffusible species—would not be able to form Turing patterns (21, 38). Indeed, of the six TGF-beta superfamily members that were dynamically expressed in our scRNA-seq pseudotime data, three were extracellular BMP inhibitors: *CHRDL1, CHRDL2,* and *NOG* [Fig. 1*E*, (39)]. Although overexpression of *CHRDL1* can cause loss of entire skeletal elements in the chicken hindlimb (40), no reported mutations in either of the two *CHRDL* genes affect joint patterning in the mouse or human. In contrast, mutations in *NOG* are frequently associated with interphalangeal joint defects in humans, and *Nog(−/−)* mice fail to develop joints (30, 41). Therefore, we hypothesized that the diffusible BMP inhibitor NOG, together with GDF5 and pSMAD, would define a minimal Turing network capable of self-organized periodic patterning, to dictate the repetitive initiation of joint interzones.

**Fig. 2.**
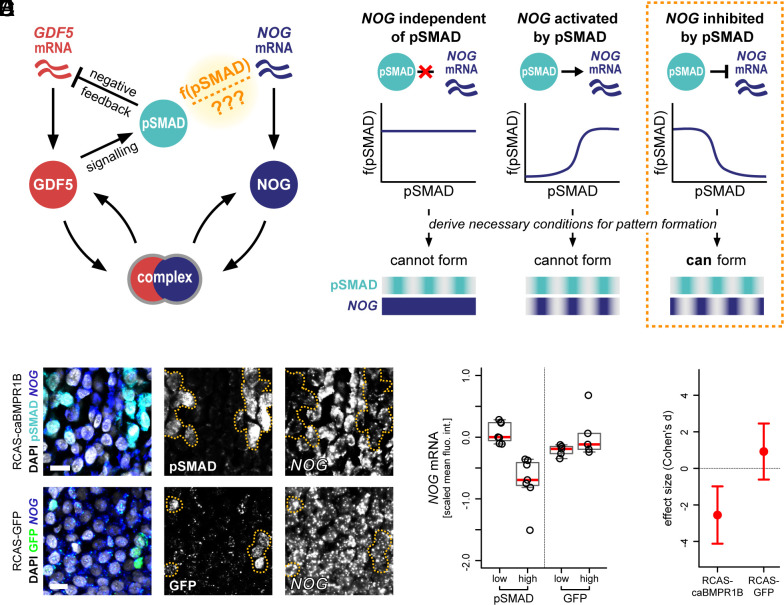
Theory predicts that BMP signaling must inhibit *NOG* expression for Turing-like joint patterning to occur. (*A*) Interactions in the BMP signaling pathway known to be involved in joint patterning: GDF5 activates BMP signaling (pSMAD) (44, 45); pSMAD negatively regulates *GDF5* transcription (16); NOG binds GDF5 to prevent it from signaling (46). It is not known how or whether active BMP signaling affects *NOG* transcription. (*B*) Mathematical modeling revealed that, regardless of biophysical parameters, self-organized periodic joint patterns can only form if *NOG* transcription is negatively regulated by active BMP signaling (see **SI Appendix*, Text S2* for proofs and linear instability analysis). (*C*) Mosaic expression of constitutively active BMPR1B (caBMPR1B, *Top*) or Green Fluorescent Protein (GFP, *Bottom*) in the distal digit domain in vivo. Ectopic induction of pSMAD leads to reduced *NOG* mRNA levels in a cell-autonomous manner, whereas GFP expression does not (clonal boundaries delineated by yellow dotted lines). (Scale bars, 10 μm.) (*D*) Quantification of *NOG* mRNA levels in control cells (“low”), or cells transfected with caBMPR1B (“high”, *Left*) or GFP (high, *Right*). Mean scaled fluorescence values are plotted for independent biological replicates. (*E*) Effect sizes (Cohen’s d) on cellular *NOG* mRNA levels for RCAS-caBMPR1B and RCAS-GFP transfections. 95% confidence intervals are indicated.

To explore this hypothesis, we formulated a mathematical model of BMP signaling dynamics in the developing digit. Our core assumptions were that 1) GDF5 activates pSMAD; 2) pSMAD inhibits *GDF5* transcription; 3) NOG binds GDF5 to form a complex; 4) the NOG-GDF5 complex cannot bind BMP receptors i.e., cannot activate signaling; and 5) NOG, GDF5 and the NOG-GDF5 complex diffuse in the extracellular space (Fig. 2*A*). Initially, we also assumed *NOG* to be expressed uniformly throughout the digit ray, based on published expression data for mouse and chicken (13, 39, 41). Together, these assumptions define a reaction–diffusion system, and we used partial differential equations (PDEs) to describe the spatiotemporal dynamics of each component (*SI Appendix*, Fig. S2*A* and *Text S1*). Using linear instability analysis (38, 42, 43), we could derive general conditions that are necessary for periodic patterns to form as expected. Surprisingly, these analyses predicted that this reaction–diffusion system was unable to self-organize patterns for any combination of parameters. We therefore revisited the core assumptions of our model, focusing on the spatiotemporal dynamics of *NOG,* for which quantitative expression data had not previously been collected. We generalized the model and now allowed *NOG* expression to vary spatiotemporally, hypothesizing that feedback regulation of *NOG* expression by pSMAD activity may be required for the network to self-organize. When we re-analyzed our model with these more general assumptions, we were now able to identify a network that could spontaneously form patterns. We find that, regardless of model parameters, a necessary condition for patterning is that pSMAD activity must down-regulate *NOG* expression (Fig. 2*B* and **SI Appendix*, Text S2*).

To test this prediction in vivo, we induced ectopic patches of pSMAD in the developing digits, using viral overexpression of a constitutively active BMPR1B receptor (caBMPR1B) (*SI Appendix*, Fig. S3*A*). Transfected cells in the distal, non-segmented digit domain displayed a cell-autonomous downregulation of *NOG* mRNA expression, something that was not apparent in control transfections expressing Green Fluorescent Protein (GFP) (Fig. 2*C*). We then systematically analyzed cells from these two experimental conditions, i.e., with or without caBMPR1B or GFP expression (*SI Appendix*, Fig. S3 *B*–*F*), and found that cells with ectopic pSMAD showed a consistent reduction in *NOG* mRNA levels (Fig. 2 *D* and *E*). Conversely, expression of GFP had a negligible impact on *NOG* transcription (Fig. 2 *D* and *E*), compatible with the transfection having no effect (Cohen’s d = 0). Together, these results confirm an inhibitory effect of pSMAD activity on *NOG* transcription in the distal digit domain, as predicted by our mathematical analysis.

A consequence of this regulatory interaction is that *NOG* must be expressed in a periodic pattern, out of phase with pSMAD activity, for Turing-like pattern formation to occur (Fig. 2*B*). This predicted pattern is in contrast to previous reports of largely homogenous *NOG* expression along the digit (13, 39, 41). Accordingly, we decided to compare the spatiotemporal in silico dynamics of our Turing model with quantitative in vivo expression data.

### A BMP-Based Turing Model Recapitulates In Vivo Expression Dynamics during Digit Patterning.

**Fig. 3.**
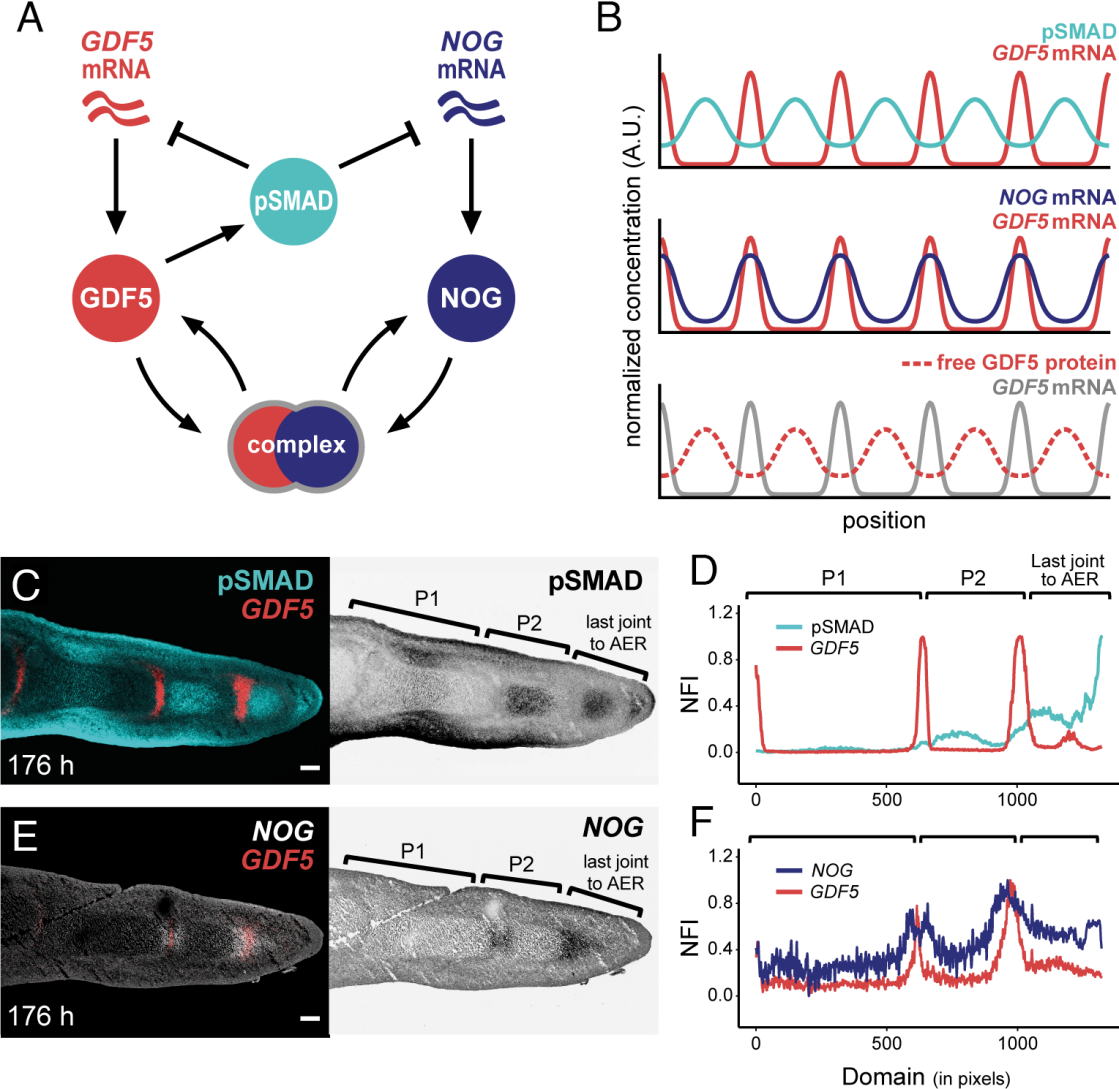
A BMP-based Turing model recapitulates in vivo signaling patterns and digit segmentation. (*A*) A BMP-based Turing model of digit patterning. (*B*) Simulated 1D expression patterns; plots show normalized expression of *GDF5* and *NOG* mRNA, pSMAD activity, and GDF5 protein dynamics along the proximal-distal digit axis. (*C*) Fluorescent RNA in situ hybridization for *GDF5* with immunohistochemistry for pSMAD on longitudinal sections of hindlimb digit III at 176 h of development. (*D*) Plot of NFI for pSMAD (cyan) and *GDF5* (red). (*E*) Fluorescent RNA in situ hybridization for *GDF5* and *NOG* on an immediately adjacent section of (*C*). (*F*) Plot of NFI for *GDF5* (red) and *NOG* (blue) along the proximal-distal domain of digit III at 176 h of development. (Scale bars, 100 μm.) 1 pixel = 1.243 μm.

We first simulated our model on a one-dimensional domain to understand its predicted dynamics, initially neglecting digit growth to focus on the intrinsic ability of the BMP network to self-organize. To satisfy the necessary condition for patterning derived above, we assumed that *NOG* expression was inhibited by BMP signaling (Fig. 3*A*). We found that this network topology spontaneously formed periodic patterns with phase differences between the model components. To visualize these patterns, we plotted the concentrations of extracellular GDF5 and NOG protein, the level of pSMAD activity, and the inferred mRNA levels of *GDF5* and *NOG* (**SI Appendix*, Text S3*). We found that *GDF5* and *NOG* mRNAs were expressed in repeating peaks that were in-phase with one other, but out of phase with pSMAD activity. At the presumptive joint regions, where both *GDF5* and *NOG* are expressed, we predicted that high levels of NOG sequester GDF5 protein into complex (*SI Appendix*, Fig. S2*B*), thereby preventing it from activating the BMP pathway. However, at a farther distance from the joints, extracellular diffusion allows unbound GDF5 to accumulate and activate BMP signaling, thus rendering the peaks of free GDF5 protein in phase with pSMAD (Fig. 3*B*). In contrast, extracellular NOG protein remains localized to the joint regions where it inhibits BMP activity (*SI Appendix*, Fig. S2*B*). Intuitively, we might expect that the difference in the spread of GDF5 and NOG from the joints would require them to have different diffusion coefficients. However, we found that these self-organizing patterns form across a wide range of parameter space and do not require differential diffusivities (*SI Appendix*, Fig. S2*C*) suggesting that other parameters are able to modulate the effective range of GDF5 and NOG. Importantly, we observed the same characteristic phase differences between model components for all parameter combinations that we tested, suggesting that this qualitative feature of the patterns is robust to parameter choice.

To compare our in silico predictions to the spatiotemporal dynamics in vivo, we next quantified expression patterns of *GDF5, NOG*, and pSMAD activity in the segmenting digits. We found that these in vivo measurements closely matched key predictions from our BMP-based Turing model. Namely, *GDF5* mRNA expression was observed in the presumptive joint regions, with periodic maxima of pSMAD concentration localized in the intervening phalangeal regions (Fig. 3 *C* and *D*). Double FISH for *GDF5* and *NOG* indicated that *NOG* mRNA expression is more dynamic than previously reported, forming a repeating pattern of peaks approximately in phase with *GDF5* mRNA (Fig. 3 *E* and *F*). Furthermore, by comparing the resulting profiles to quantifications of *GDF5* and pSMAD on adjacent sections, we found that *NOG* peaks were predominantly out of phase with pSMAD activity (Fig. 3 *C*–*F*).

While our model correctly predicted the relative phases of *GDF5*, *NOG,* and pSMAD, so far, it simulated patterning on a static domain. To explain the temporal progression of patterning observed within an elongating digit in vivo, we incorporated growth dynamics into our simulations, assuming that 1) digits grow primarily at their distal tip, via the incorporation of uncommitted sub-AER progenitors into the PFR; 2) cells irreversibly commit to either the joint or phalanx fate at a certain distance away from the PFR; and 3) patterning within the uncommitted distal digit domain is described by our GDF5/NOG/pSMAD Turing model. We also added an additional source of BMP activity at the distal digit tip, in order to mimic the high levels of pSMAD observed at the PFR (**SI Appendix*, Text S3*). In vivo, elevated pSMAD levels may result from a combination of factors, including localized *Activin* signaling at the digit tip (7), BMPs expressed in the interdigital mesenchyme (6, 11, 13, 47), and/or mechanical interactions at the boundary between the PFR and the digit (10). Taken together, this set of model assumptions significantly oversimplifies the complex growth and differentiation dynamics that are likely to be operating in vivo; nonetheless, it may provide insight into the potential impact of digit growth on phalanx-joint patterning. When we included growth and cell fate commitment in our simulations, we observed the same phase differences as before, but now, instead of forming simultaneously, *GDF5* peaks appeared sequentially. New joints formed toward the tip of the elongating digit, matching their iterative dynamics observed in vivo (*SI Appendix*, Fig. S4*A*). Similar dynamics were observed for pSMAD activity, but with opposite phase, accompanying the periodic formation of sequential phalanges both in silico and in vivo (13) (*SI Appendix*, Fig. S4 *A* and *B*).

Importantly, the earliest evidence of symmetry breaking in the distal patterning domain closely matched our in silico predictions, with a drop in pSMAD activity coinciding with the initiation of *GDF5* mRNA expression (*SI Appendix*, Fig. S4 *C* and *D*, arrows). To quantify these patterns further, we assembled combined measurements for either *GDF5*/pSMAD or *GDF5*/*NOG*, from multiple embryos and time points, focusing on the distal end of each digit where the earliest patterning events occur as cells exit the PFR (*SI Appendix*, Fig. S5 *A* and *B*). By using the two consecutive peaks of *GDF5* expression in this distal domain as landmarks, i.e., demarcating the previously formed joint and the newly emerging one, we aligned fluorescence intensity profiles from multiple, individual embryos onto the same spatial axis. We observed that superimposed profiles of both *GDF5* and *NOG* expression peaked around the newly forming joint and that pSMAD activity was in near-perfect antiphase to them (*SI Appendix*, Fig. S5*C*). Notably, the more pronounced and wider peak of *NOG* expression, compared to *GDF5*, was in agreement with our in silico simulations (*SI Appendix*, Fig. S5*D*), and the predicted *GDF5*/*NOG* co-expression was recapitulated at the cellular level in our scRNA-seq data (*SI Appendix*, Fig. S5 *E* and *F* and Fig.1*E*). These observations thus corroborated that the early, distal dynamics of patterning in vivo are consistent with the predictions from our BMP-based Turing model.

Quantifying expression profiles in more proximal digit regions, in which both phalanges-flanking joint interzones had already been specified, revealed alterations to the BMP signaling dynamics. Namely, profiles of both pSMAD and *NOG* evolved further, relative to the two flanking *GDF5* peaks marking the proximal and distal joints (*SI Appendix*, Fig. S6*A*). The peak of the pSMAD shoulder shifted proximally, and its minimum no longer coincided with the distal joint, leading to a relative phase-shift and thus asymmetry between the *GDF5* and pSMAD patterns. *GDF5* expression at the distal joint increased, and its domain sharpened (*SI Appendix*, Fig. S6*A*). Furthermore, *NOG* expression continued to be expressed largely out of phase with pSMAD but was excluded from the joint itself. Namely, the peak of *NOG* expression split, with the two resulting maxima now flanking the peaks of *GDF5*, as evidenced in embryos with slight developmental heterochronies between them [*SI Appendix*, Fig. S6*B*, see also Fig. 3*F* [P1/P2 transition], and (16)]. Importantly, however, these dynamics all occurred after the initial, segmentation-relevant symmetry breaking, and thus were not accounted for by our model.

Collectively, by combining in vivo data with in silico simulations, we found evidence for a BMP-based Turing system that underlies iterative phalanx-joint pattering during digit elongation. We also confirmed a key prediction of our model, namely that *NOG* should be expressed in a periodic pattern, out of phase with pSMAD.

### Spatiotemporal BMP Signaling Dynamics Are Evolutionarily Conserved and Predictive of Known Patterning Perturbations.

**Fig. 4.**
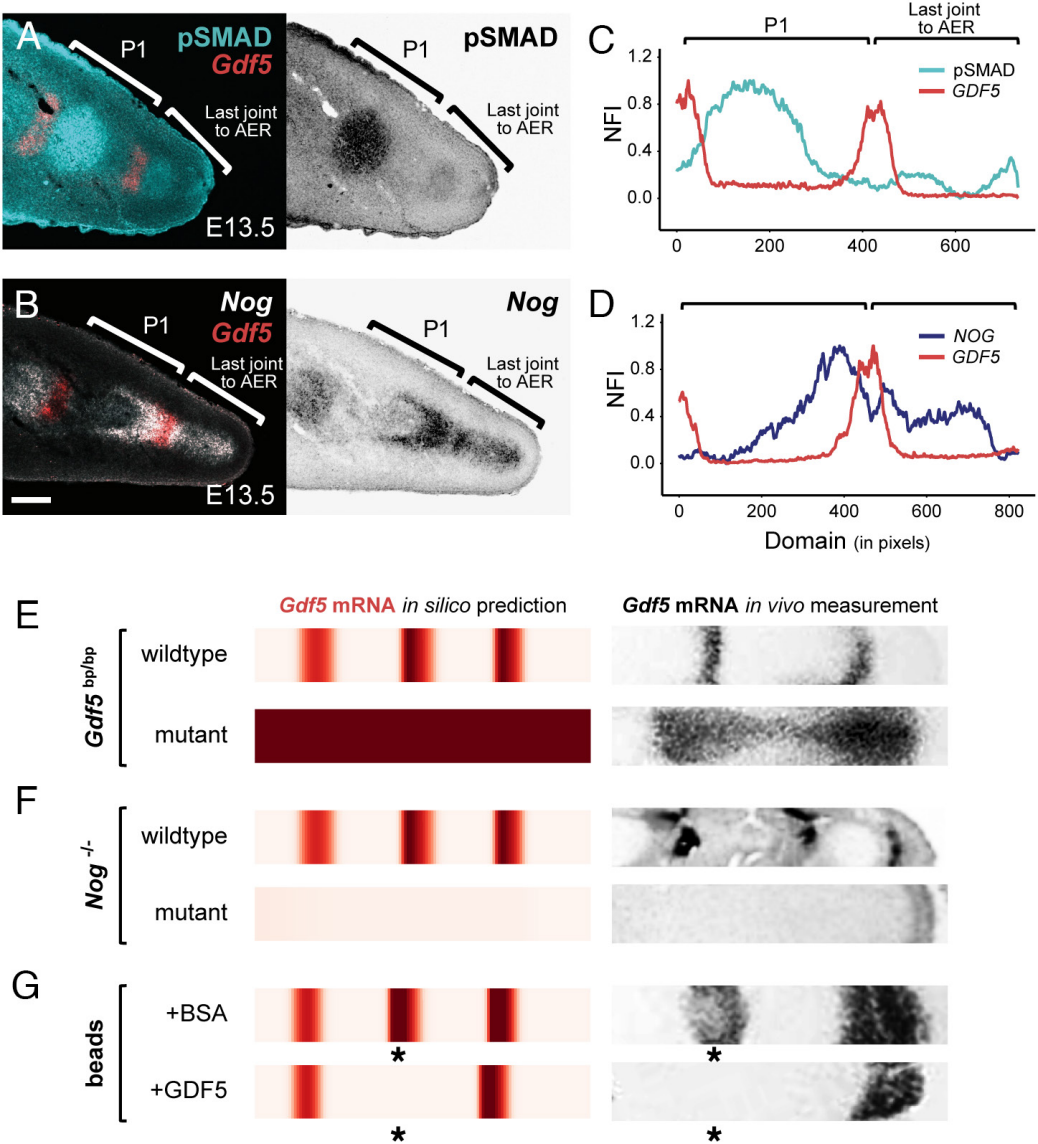
Conserved self-organized BMP signaling dynamics underlie mouse digit patterning and perturbation phenotypes (*A* and *B*) Fluorescent RNA in situ hybridization for *Gdf5* combined with immunohistochemistry for pSMAD (*A*) or in situ hybridization for *Nog* (*B*) on immediately adjacent sections of mouse digit 2 at E13.5. (*C* and *D*) Plots of NFI of pSMAD (cyan) and *Gdf5* (red) (*C*) and *Gdf5* (red) and *Nog* (blue) (*D*). (Scale bar, 100 μm.) 1 pixel = 1.243 μm. (*E*–*G*) Heatmap visualizations of in silico simulations to contrast *Gdf5* wildtype expression patterns with a *Gdf5^bp/bp^* (*brachypodism*) mutant background (*E*), a *Nog* mutant background (*F*), or a wildtype background with beads soaked in either BSA or recombinant GDF5 protein (bead positions marked by an asterisk) (*G*). Corresponding in vivo *Gdf5* in situ hybridization images are provided in black-and-white (*E* and G^*^; *F*^†^).

A repeating pattern of joints is a hallmark characteristic of digits across the tetrapod clade. Therefore, we wondered whether the self-organizing BMP network we identified in chicken could also be operating in other, distantly related species, to drive periodic joint patterns. To explore this, we examined BMP signaling dynamics during mouse digit development. We performed FISH for *Gdf5* combined with IHC for pSMAD on longitudinal digit sections at embryonic day E13.5, as well as double FISH for *Gdf5* and *Nog* on adjacent sections (Fig. 4 *A* and *B*). Line plot quantifications of NFI revealed that, indeed, similar expression dynamics were occurring during early mouse digit segmentation. Namely, while peaks of *Gdf5* expression were out of phase with pSMAD activity, *Nog* and *Gdf5* mRNA profiles were largely in phase with one another (Fig. 4 *C* and *D*). As in chicken, we observed a bimodal peak of *Nog* expression, centered on an already maturing *Gdf5*-positive joint interzone (Fig. 4*D*), with the maximal levels of *Nog* being expressed out of phase with pSMAD (Fig. 4, compare *Nog* to pSMAD in *D* and *C*). Peaks of pSMAD appeared to shift toward the proximal *Gdf5* bands (Fig. 4*C*), although based on our more detailed time series in the chicken, we hypothesize that this asymmetry is associated with phalanx maturation (*SI Appendix*, Fig. S6 *A* and *B*). We thus concluded that the spatiotemporal dynamics of BMP pathway members during early digit patterning are largely conserved between birds and mammals. This suggests that the same core Turing system—involving GDF5, NOG, and pSMAD—could be responsible for determining the periodicity of interphalangeal joint segmentation patterns across amniotes.

We then took advantage of molecular genetics studies in mice that have described joint patterning defects caused by mutations in the main components of our model. By changing model parameters, we tested whether mimicking genetic perturbations in silico would correctly predict the resulting effects on *Gdf5* expression, an early marker of joint specification. First, we considered mutants in which GDF5 cannot activate BMP signaling. In this case, our model predicted *Gdf5* mRNA to be uniformly expressed at a high level throughout the simulated digit, instead of the characteristic repeating stripes. Such a pattern of *Gdf5* expression has indeed been reported in embryos carrying mutations disrupting the GDF5 coding sequence (Fig. 4*E*, *brachypodism*) (27, 48), or lacking its essential signaling partner BMPR1B (49). Although this change in *Gdf5* transcription may appear counterintuitive—since inactivating an interzone marker led to an apparent expansion of the interzone domain—it is a natural consequence of the Turing-like negative feedback logic central to our model. Second, simulating mutants in which NOG is unable to inhibit GDF5 signaling activity predicted a downregulation of *Gdf5* expression and loss of its periodic pattern. Indeed, *Nog(−/−)* mutants fail to express *Gdf5* in digits, and do not develop interphalangeal joints (41) (Fig. 4*F*). Finally, we considered a spatially restricted perturbation to digit patterning, by mimicking the effect of an implanted GDF5-soaked bead. This led to the local inhibition of *Gdf5* mRNA expression both in silico and in vivo (27) (Fig. 4*G*, asterisks). Taken together, the ability of a single model to explain the expression dynamics and mutant phenotypes in both mouse and chick hints at a conserved BMP-based Turing mechanism that is responsible for digit segmentation patterns in birds and mammals.

### Segmentation and Growth Dynamics in Two Morphologically Distinct Digits.

**Fig. 5.**
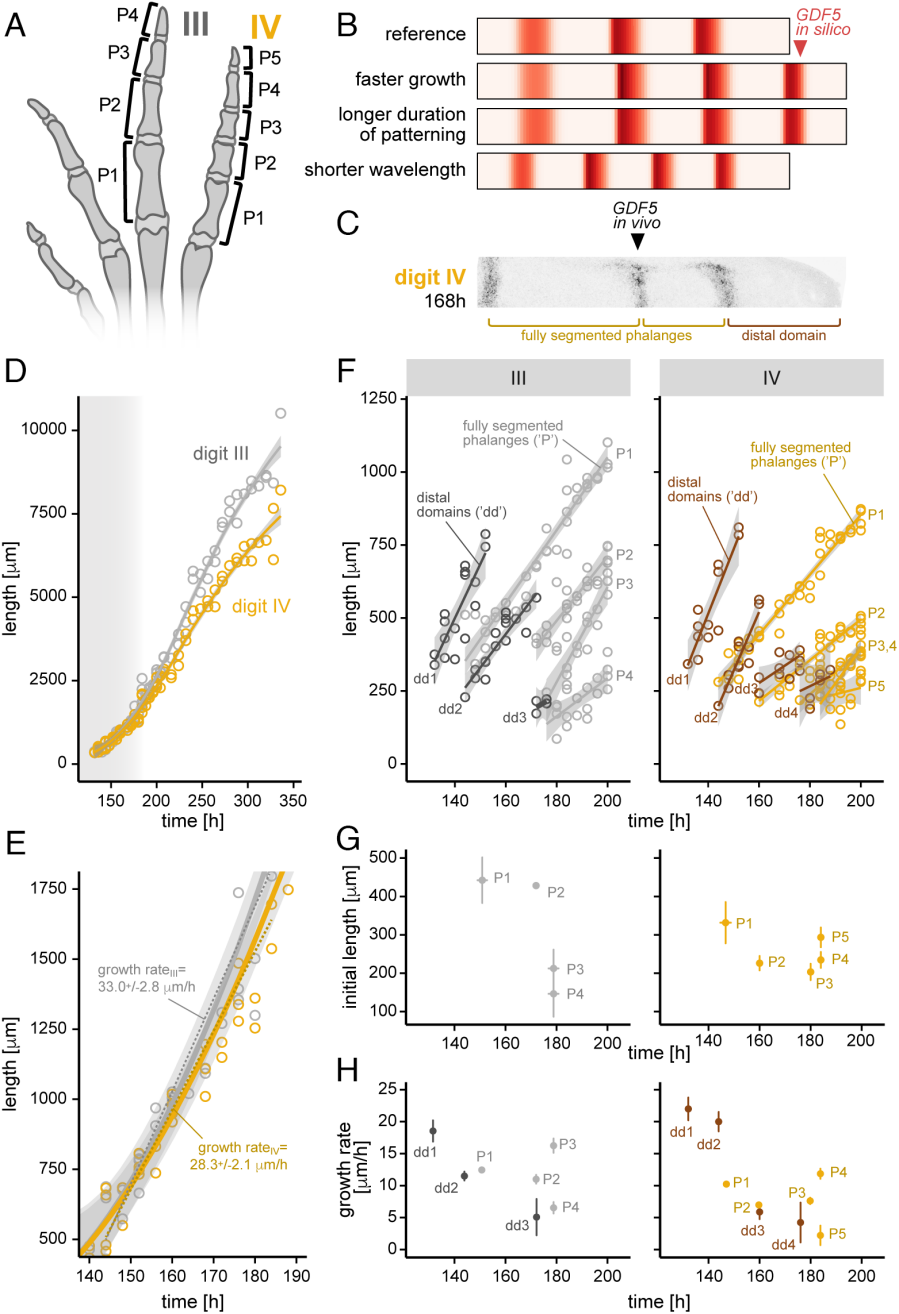
Growth and segmentation of two morphologically distinct digits of the developing chicken hindlimb. (*A*) Phalangeal morphologies of digits III and IV in the chicken foot. (*B*) In silico modeling predicts that an additional phalanx-joint element (=extra *GDF5* band) can form due to faster growth, longer duration of patterning phase, or a shorter Turing wavelength. (*C*) Longitudinal section of digit IV, stained for *GDF5*. (*D*) Total digit growth dynamics for digits III and IV. The shaded gray area approximates the time window of digit segmentation. (*E*) Zoom-in for the phase of digit segmentation. A linear model of digit elongation for digits III and IV, from 144 h to 184 h, is superimposed to approximate the respective growth rates. (*F*) Growth and segmentation dynamics of individual elements for digits III and IV. Linear models of each element’s growth are plotted in either dark, for the distal domains (“dd”) spanning from the last formed joint to the digit tip, or light colors, for fully segmented phalanges (“P”). (*G*) Initial mean length and first developmental appearance for the different phalanges of digits III and IV. (*H*) Initial growth rates of the different digit segments, either spanning from the last formed joint to the digit tip (dd) or fully segmented phalanges (P) in digits III and IV.

Phalangeal digit patterns display a large range of morphological diversity across amniotes, with the size and number of skeletal elements varying considerably both within and between species. We used our BMP-based Turing model to investigate the potential causes of these variations. As an in vivo model system to compare against, we took advantage of the chicken foot, in which each digit shows a distinct phalangeal formula. For example, in its adult form, digit III is longer than digit IV, but contains one fewer phalanx (Fig. 5*A*).

We began by using our in silico model to explore different scenarios that could account for the observed differences in phalanx numbers. First, we found that increasing the total length of the segmenting digit domain, either through faster growth or a prolonged window for patterning, led to additional *GDF5* bands and hence the appearance of a supernumerary phalanx (Fig. 5*B*, faster growth or longer duration). Alternatively, if the underlying wavelength of the Turing system was reduced, an additional skeletal element could be produced without changing digit length (Fig. 5*B*, shorter wavelength). Indeed, we found that variations to several parameters in our model could result in significant changes to the pattern wavelength (*SI Appendix*, Fig. S7 *A* and *B*). For example, doubling the rate at which extracellular NOG was removed from the system sufficed to generate an extra phalanx in silico (Fig. 5*B*, shorter wavelength).

To discriminate between these different scenarios, we produced quantitative growth and segmentation data for digits III and IV along a developmental time series (from 128 h to 336 h; roughly HH27-HH40). Using longitudinal digit cryosections to identify consecutive bands of *GDF5* expression, we measured the entire length of the digit, defined from the metatarsophalangeal joint to the AER; the lengths of individual, fully segmented phalanges; and the lengths of the unsegmented distal domain, defined from the most distal joint interzone to the AER (Fig. 5*C*, see *Materials and Methods* for details). Looking at overall digit growth over time (Fig. 5*D*), we saw that the major differences in digit lengths between digit III and IV only appeared after digit segmentation had completed (≈180 h). During active digit segmentation (≈140 to 180 h), growth rates of digits III and IV were nearly indistinguishable (Fig. 5*E*). We thus excluded overall faster growth as a possible explanation for the formation of an additional, fifth phalanx in digit IV.

To investigate potential digit-specific differences in patterning duration and/or wavelength, we next plotted the dynamics of each element of the two digits individually. Again, we discriminated between fully segmented phalanges (light colors) and the unsegmented distal domains (dark colors, Fig. 5*F*). As expected, joint interzones initiated sequentially as the digit elongated, to drive periodic phalanx segmentation (13). However, both within as well as across the two digits, the size, growth rates, and time of initiation varied between the different elements. Digit segmentation in digit III appeared to complete slightly earlier (before 180 h) than in digit IV (after 180 h) (Fig. 5 *F* and *G*). This difference in timing, however, appeared insufficient to fully account for the growth of an entire extra phalanx, compared to the durations seen for previously formed ones [≈15 to 20 h, (Fig. 5*G*)]. We therefore investigated at which length each newly forming phalanx was individualized, i.e., was demarcated by the appearance of a faint *GDF5* band delineating its distal end (Fig. 5*G*). This initial length serves as a proxy for the segmentation wavelength in our in silico model, since it corresponds to the early patterning prior to subsequent growth. In both digits, distal phalanges showed a tendency to be progressively smaller at their onset and were initiated at shorter time intervals. Importantly, however, up to the penultimate elements, phalanges in digit III (P1_III_ and P2_III_) were longer at their point of initiation compared to their digit IV counterparts (P1_IV_, P2_IV_, and P3_IV_). This suggested that the extra phalanx in digit IV may be, in part, attributed to a shorter wavelength of the underlying Turing system.

Finally, to explore the effect of growth on phalangeal proportions, we quantified the growth rates for each individual phalanx, as well as the unsegmented distal domains, and contrasted them across the two digits (Fig. 5*H*). In both digits, most individual growth rates appeared to decline over developmental time, with the distal domains showing a steeper decline than the fully segmented phalanges. A notable exception to this trend were the two penultimate elements. In both digits, these phalanges (P3_III_, P4_IV_) showed a marked increase in their growth rates relative to the other digit elements. This highlights how variations to post-segmentation growth may influence the final morphology of the digit independently of early patterning.

Overall, our results reveal differences in growth and segmentation dynamics between two morphologically distinct digits. We find variations in phalanx size, both within as well as across the two digits, and show how these may be caused by alterations to phalanx-specific growth rates and/or to the reaction–diffusion parameters of the underlying Turing mechanism.

## Discussion

Digits within and across species vary in shape and size, due to changes in the numbers and the dimensions of their individual digit bones, the phalanges. Many of the morphological extremes relate to adaptations toward distinct modes of locomotion, such as the elongated phalanges in the digits of a bat wing, or the hyperphalangy with more than 10 individual skeletal elements per digit in the flippers of certain whales (4, 14). Here, we have studied the early developmental basis for this morphological diversity, focusing on the molecular and cellular dynamics giving rise to distinct digit segmentation patterns. Combining quantitative in vivo data with in silico simulations, we present a model in which the segmentation wavelength is determined by a Turing-like mechanism involving the BMP-ligand GDF5 and its extracellular inhibitor NOG. Furthermore, distinct growth rates of individual elements, within and between digits, seem to have enabled a highly modular approach to diversifying digit morphologies in terms of phalanx lengths and numbers.

Based on pseudotemporal ordering of scRNA-seq transcriptomic data and in vivo quantifications of BMP signaling dynamics, we propose GDF5—a BMP-activating ligand expressed and secreted at forming joint sites—as the inhibitor of a Turing-like reaction–diffusion system. Furthermore, theory and in vivo data suggest that highly dynamic *NOG* expression, in phase with *GDF5*, completes this early self-organizing process to specify joint interzone locations. By modeling the reaction–diffusion dynamics of *GDF5* and *NOG* in silico, we predict that *NOG* must be expressed out of phase with pSMAD for the system to organize into repetitive patterns. While this notion was at odds with previous *NOG* expression studies, it was nonetheless supported by our quantitative FISH measurements and the negative impact of ectopically induced pSMAD on *NOG* transcription in the distal digit domain. Importantly, we find that the transcriptional signatures and BMP signaling dynamics predicted by our model closely match quantitative expression data from developing digits, in both chicken (Fig. 3) and mouse (Fig. 4). Moreover, the model successfully phenocopies aberrant digit patterns resulting from perturbations to the BMP pathway in mouse. Similar digit malformations are observed in humans, with analogous genetic alterations in *GDF5, NOG,* or *BMPR1B* commonly associated with digit patterning defects (30). Our model can therefore explain the etiology of congenital human conditions in which the digits lack either interphalangeal joints [symphalangism (50)] or phalanges [Chondrodysplasia Grebe type (51)]. Collectively, these results suggest that a conserved BMP-based Turing mechanism is responsible for digit segmentation patterns across amniotes.

Central to this Turing mechanism is the differential spread of GDF5 and NOG proteins away from their overlapping domains of expression, allowing NOG to inhibit the BMP pathway at the initiating joint region, and GDF5 to activate pSMAD at a distance. Our simulations show that this can be achieved across a wide range of parameter sets—something which is increasingly being observed in Turing models (38, 52, 53)—and does not necessarily require differential diffusivity between GDF5 and NOG (*SI Appendix*, Fig. S2*C*), although further work would be needed to comprehensively characterize the relevant parameter space. Moreover, while many parameters are compatible with patterning in silico, measurements of key biophysical properties (e.g., diffusion coefficients, degradation rates) will be required to determine the relevant regime in vivo. For example, we hypothesize that extracellular NOG has a low diffusivity, which could potentially be explained by its strong affinity to heparin sulfate (54, 55). Indeed, this binding appears to be important for patterning since mutations in the heparin-binding site of NOG (56) or modifications to heparin sulfate itself (57) lead to aberrant joint patterns. While such parameter values have yet to be experimentally quantified, our theory (**SI Appendix*, Text S2*) and simulations (*SI Appendix*, Fig. S2) show that qualitative predictions from the model apply broadly across parameter space.

An intriguing feature of our model is its simplicity, with the initial segmentation patterns driven by a single developmental signaling pathway. Undoubtedly, specifying distinct digit morphologies in vivo will involve multiple other signaling pathways, as well as more complex morphogenetic processes than we have approximated in silico. These are likely to include changes in cell shape and adhesion, integration of mechanical forces, and differential regulation of long bone growth (10, 58, 59). However, by considering the BMP pathway alone, we were still able to capture the earliest expression dynamics associated with digit segmentation and predict key mutant phenotypes. Moreover, genetic evidence from the mouse implies that other signaling pathways known to affect digit patterning are likely dispensable in the core Turing network for the periodic placement of joint interzones [see e.g., WNTs (60, 61), Hedgehog signaling (13, 62), extradigital BMPs (63, 64)]. This suggests that interactions between GDF5, NOG, and pSMAD may be sufficient for the initial symmetry breaking. However, we do not exclude the possibility that other signals—such as AER-derived FGFs or extradigital BMP/TGF-beta ligands—might modulate the segmentation dynamics, especially in the distal digit regions. FGF signaling, in particular, may play a dual role here, by affecting the rate and duration of digit ray elongation—and, hence, the number of phalanges (11, 14)— as well as tuning BMP responsiveness through regulation of BMPR1B expression (65), which may influence the initial wavelength of joint spacing.

A further simplification of our model is that we consider patterning only within a one-dimensional (1D) domain, rather than the full 3D geometry of the developing digits. The predictions in Fig. 2 hold regardless of dimension, meaning our model will form periodic patterns with the same overall phases in 2D or 3D as we here presented for 1D. Nevertheless, beyond the earliest symmetry breaking events, we expect that other pathways will be involved in refining a 3D digit pattern. In particular, we hypothesize that while our BMP-based model can initiate repetitive patterns with periodic dots of pSMAD activity, other pathways—or BMP signaling components—will be required to sharpen the initially broad domains of *GDF5* into straight, narrow stripes (18). Hedgehog signaling is a promising candidate for future investigation since *Gdf5* bands remain broad and curved in *Gli3*(−/−) digits (13). Furthermore, *CHRDL1* and *CHRDL2*, with their complementary patterns in the phalanx and joint domains, respectively, may also contribute to refine the stripe-like pattern of late *GDF5* expression (Fig. 1*E*). And finally, while BMPR1B is expressed throughout the distal digit (7), thus rendering a ligand–receptor-based Turing mechanism unlikely (66), subsequent regulation of BMPR1B levels may contribute to the maturing *GDF5* pattern. Indeed, the exclusion of *BMPR1B* expression from the maturing joint itself, and the importance of *BMPR1A* at later stages, may reflect a transition into a new BMP signaling regime [Fig. 1*E*, (7, 67, 68)].

*NOG* and pSMAD both show changes in their expression and activity patterns that are likely related to a switch in the predominant mode of phalanx elongation, transitioning from mostly distal progenitor proliferation to epiphyseal plate-driven long bone growth. In proximal phalanges, we see a phase shift of the pSMAD pattern relative to the *Gdf5* peaks, with the pSMAD peaks moving toward the proximal joint, and an increase of pSMAD being observed near the distal joint (*SI Appendix*, Fig. S6*A*). During long bone development, two peaks of pSMAD activity mark the distal growth zones at either end of the skeletal element. These pSMAD domains rely on BMPs from the perichondrium and the epiphyseal plate itself and are essential for signaling cross talk and endochondral bone elongation (69). Furthermore, two defined bands of NOG flanking the interzone safeguard continuing joint formation against these BMP signals (16, 70, 71). This split in the early interzone-centered domain of *NOG* expression seems to evolve as the forming phalanges transition from distal domain segmentation to long bone growth (*SI Appendix*, Fig. S6*B*).

Like for the observed BMP signaling dynamics, the presence of two distinct temporal regimes—i.e., pre- and post-segmentation—also manifests itself in digit growth, both within and across digits. Changes in total digit lengths within the same autopod appear to largely arise during the post-segmentation phase (Fig. 5*D*). Before that, however, growth rates already differ among individual phalangeal elements within a digit, highlighting the modular nature of digit elongation [Fig. 5 *F*–*H*, (22)]. For most phalanges, these growth rates progressively decline as development proceeds (Fig. 5*H*). This occurs to a similar extent in both digits III and IV, and likely relates to the waning of FGF signals from the AER, and—potentially—its impact on *BMPR1B* transcription in the distal patterning domain (11, 65). Strikingly, however, once the penultimate phalanges have formed, they show a pronounced up-tick in growth (P3_III_, P4_IV_; Fig. 5*H*). While the molecular and cellular underpinnings of this acceleration remain unknown, nature seems to have exploited this modular mode of growth regulation in the evolution of elongated, penultimate phalanges in the feet of raptors (22). Specification of the last phalanx—known to develop differently from the rest (11, 72)—might additionally alter the segmentation dynamics at the distal end of the digit.

Comparing across digits, the elements in digit IV initiate at a shorter wavelength than in digit III, for all phalanges up to the penultimate one (Fig. 5*G*). It is tempting to speculate that variations in BMP activity across the anteroposterior axis of the limb might provide a mechanistic link between our Turing model and morphologically distinct digit identities (6, 28, 47, 73). For example, BMPs from the interdigital mesenchyme may diffuse into the distal digit where they can then bind and thereby remove NOG from the system, a scenario which is predicted to decrease the segmentation wavelength (Fig. 5*B* and *SI Appendix*, Fig. S7*B*). However, known regulatory interactions between mesenchymal BMPs and FGFs from the AER, at the distal margin of the autopod, make it difficult to experimentally discriminate between changes in segmentation wavelengths and altered digit elongation dynamics (47, 74). BMP signaling may also be modulated over time as development proceeds, which may lead to dynamically changing segmentation wavelengths and therefore non-uniform phalanx sizes within a single digit (Fig. 5*G*). Ultimately, though, the most extreme variations in digit morphologies have arisen between different species, from either alterations in post-patterning growth—such as for the elongated digits in bats (75)— or a prolonged patterning window of digit growth and segmentation, as proposed for cetacean hyperphalangy (74, 76). The observed differences in segmentation wavelengths in our chicken data might thus not be due to any particular adaptive trait. Rather, they may relate to an ancient developmental constraint, due to molecular interactions between the anterior–posterior patterning system of the limb and the BMP signaling pathway (13), which manifested itself already in the ancestral condition of tetrapod digit formulas (77, 78).

Collectively, our work has identified a conserved BMP-based Turing system that is involved in the formation of the repetitive joint segmentation patterns that characterize developing amniote digits. Furthermore, by combining quantitative in vivo data with in silico simulations, we explore how variations in growth and patterning dynamics can give rise to highly distinct digit morphologies, and demonstrate how modulation of a self-organizing segmentation wavelength can alter the number of individual elements—here, the phalanges—in a repetitive pattern.

## Materials and Methods

For a detailed description, please refer to *SI Appendix*.

### Pseudotime Analyses.

Pseudotime analysis was performed on three skeletogenic clusters from a ~HH29 autopod single-cell RNA sequencing dataset (see ref. 31 for details). R packages Seurat v3.1.4 (79), Destiny (33), Slingshot (34), and MAST (80) were used for analyses, and results were visualized in RStudio.

### Embryo Tissue Sampling and Processing.

Chicken embryos were staged according to Hamburger–Hamilton (32). Mouse embryos were isolated by M. Luxey in accordance with national laws and experimental procedures approved by the Regional Commission on Animal Experimentation and the Cantonal Veterinary Office of the city of Basel (license 1951 to Rolf Zeller and Aimee Zuniga). Tissue was fixed with 4% paraformaldehyde and processed for cryosectioning.

### Immunohistochemistry.

pSMAD signal was visualized using antigen retrieval and a primary antibody against pSMAD1,5,9 (Cell Signaling 13820S, rabbit, 1:300), followed by streptavidin-conjugated peroxidase-based signal amplification using the TSA Plus Cyanine-3 or -5 kits (Akoya Biosciences).

### FISH.

In situ hybridization was carried out using standard protocols (77) and visualized with anti-digoxigenin or anti-fluorescein-POD antibodies (Roche, 1:300), followed by signal amplification using TSA Plus Cyanine-3 or -5 (Akoya Biosciences).

### NFI Measurements.

Fluorescent signals were imaged on a confocal microscope (Olympus Fluoview FV3000). Image processing and fluorescence intensities measurements were performed in Fiji, followed by data normalization and visualization in R studio.

### Mathematical Modeling.

We constructed a reaction–diffusion model of BMP signaling in the developing digit. We used systems of PDEs to describe these dynamics, with molecular interactions as schematized by Figs. 2*A* and 3*A*. These PDEs were first analyzed using linear instability analysis to derive necessary conditions for pattern formation to occur. We then performed 1D simulations using custom MATLAB scripts. Full details of the modeling are provided in the *SI Appendix, Theory Supplement*.

### RCAS-caBMPR1B Overexpression Experiments.

We used RCAS viral vectors to ectopically express a constitutively active version of BMPR1B (81) or GFP. Viral infection was verified using IHC against pSMAD or GFP (Abcam, ab13970, 1:1,000), and the viral *gag* protein (AMV-3C2, DSHB, 1:30). We quantified *NOG* FISH signals and protein marker expression using CellProfiler (82), and binarized cells into marker “ON/OFF” states using the R package segmented (83). Effect sizes and CI were calculated with the R package effsize (84).

### Length Measurements of Individual Digit Elements.

We collected chicken hindlimbs along a developmental time series ranging from 128 h to 336 h of development. *GDF5* in situ hybridization or DAPI stains were used to determine joint locations, and individual phalanx lengths were measured in Fiji. Data analysis and visualization was done in RStudio.

## Supplementary Material

Appendix 01 (PDF)

## Data Availability

MATLAB scripts have been deposited in Github: https://github.com/twhiscock/bmp_turing_joint_patterning (85). Previously published data were used for this work (GEO GSE130439) (31).

## Footnotes

^*^Reprinted from Developmental Biology 209(1), ref. 27 with permission from Elsevier.

^†^From ref. 41. Reprinted with permission from AAAS.
