# Supplementary material for: Self-organized BMP signaling dynamics underlie the development and evolution of digit segmentation patterns in birds and mammals
Source: Proc Natl Acad Sci U S A. 2024 Jan 4;121(2):e2304470121. doi: 10.1073/pnas.2304470121 (PMC10786279; doi:10.1073/pnas.2304470121)
Supplement: Supplementary file 1 — Appendix 01 (PDF) [file pnas.2304470121.sapp.pdf]

Supporting Information

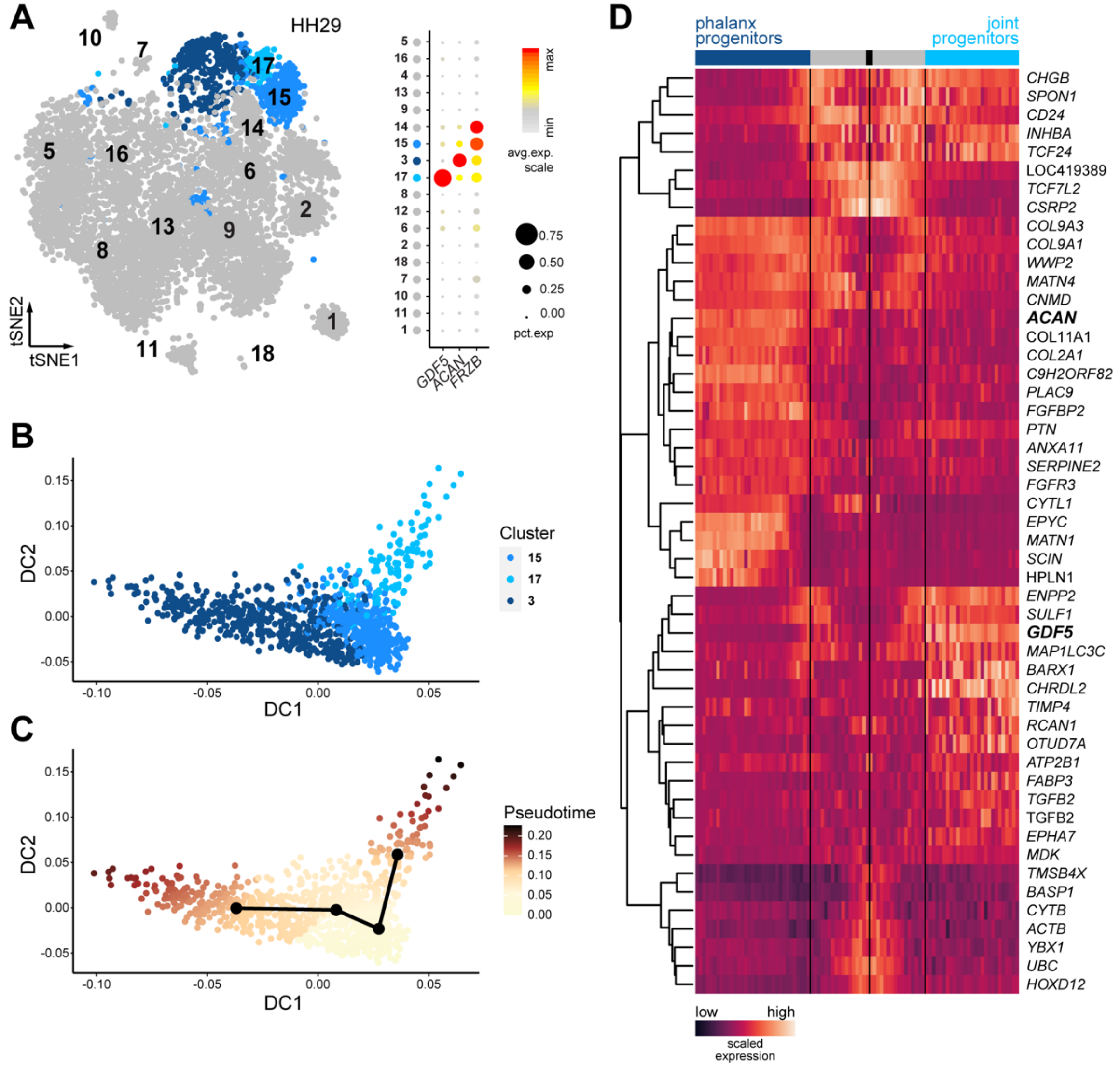

Figure S1. Single-cell pseudotemporal reconstruction of a bifurcating cell fate decision into phalanx or joint progenitor cells. (**A**) tSNE representation of a HH29 chicken hindlimb scRNA-seq dataset. Based on marker gene expression, cluster 3, 15 and 17 were selected for pseudotime analysis, to represent maturing phalanx progenitors (3), naïve skeletal progenitors and mesenchymal cells (15) and joint progenitors (17), respectively. (**B**, **C**) Diffusion map of the three clusters and their relative cellular contributions (**B**), as well as overall pseudotime progression along two bifurcating trajectories (**C**). (**D**) Pseudotime heatmap of top differentially expressed genes between the phalanx progenitor trajectory (dark blue) and the joint progenitor trajectory (turquoise). The black box corresponds to the starting point of the pseudotime, the grey zone to the part shared by the two trajectories. Scaled gene expression from low (purple) to high (orange). Non-italic names correspond to proteins identified by manual *blastx* using the cDNA sequences of the respective ENSGAL IDs.

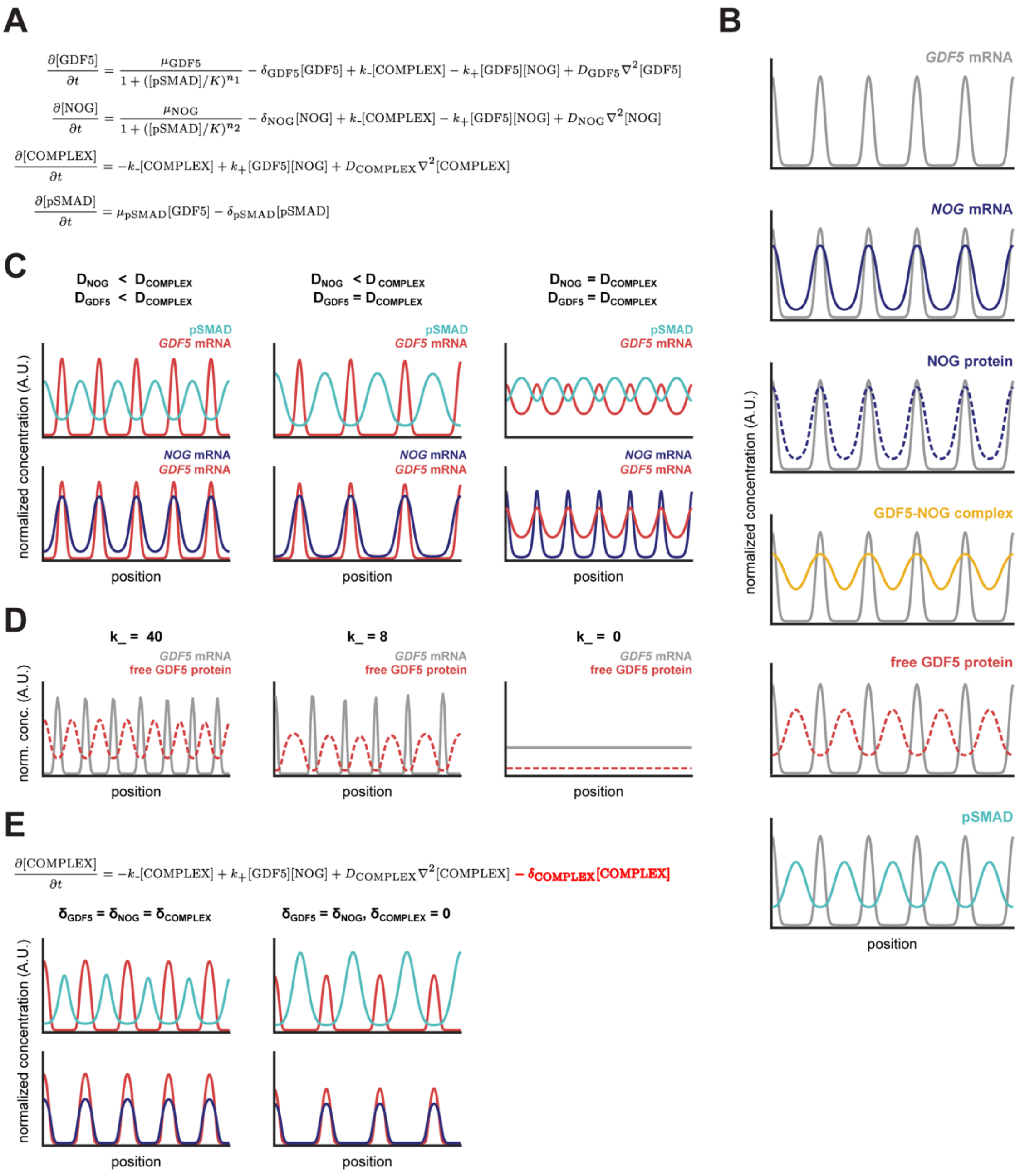

Figure S2. A mathematical model of self-organized BMP signaling in the developing digits (**A**) Partial differential equations describing the spatiotemporal dynamics of BMP signaling in the developing digits. See *SI Text S1* for more details on the model. (**B**) Predicted expression patterns shown for each of the species in the model. (**C**) Many different parameter combinations generate self-organized periodic patterns *in silico*. Here are shown three example parameter sets, with rapid complex diffusion (left); slow NOG diffusion (middle); and no differential diffusivity (right). (**D**) Predicted patterns of *GDF5* mRNA expression and "free" GDF5 protein for different values of the complex dissociation constant. (**E**) Periodic patterns can still form when assuming non-zero values for the complex degradation rate (see also *SI Text S2).*

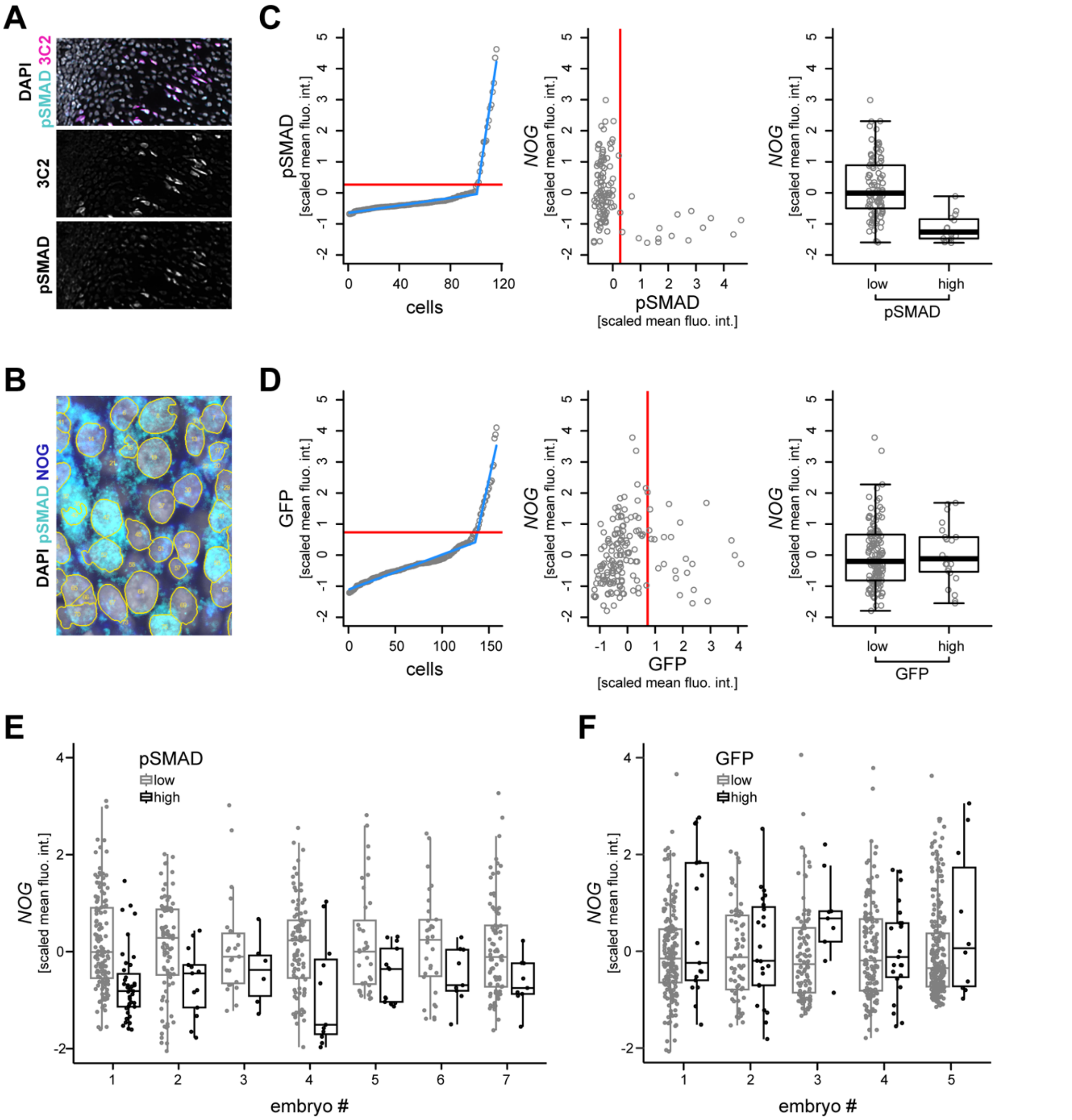

Figure S3. Ectopic activation of BMP signaling in cells of the distal digit domain and quantification of *NOG* mRNA levels (**A**) Transfection of cells with a retroviral vector expressing a constitutively active BMPR1B receptor leads to the cell-autonomous induction of pSMAD. Cellular infections are verified by IHC against the viral *gag* protein (3C2 staining). (**B**) Representative image from our *CellProfiler* image analysis pipeline. Based on the DAPI channel, masks (=yellow) are identified in which mean fluorescence intensities are quantified for *NOG* mRNA and either pSMAD or GFP protein levels. (**C**, **D**) Analysis of single representative images of stained sections from embryos transfected with either RCAS-caBMPR1B (**C**) or RCAS-GFP (**D**). Cells are categorized into either ‘low’ or ‘high’ (see red line cut-off), according to the measured values of pSMAD or GFP, based on a ‘broken line regression’ analysis (=blue line) of the respective marker intensities. The corresponding *NOG* mRNA levels are then visualized in a scatterplot, and quantified according to the two categories in a boxplot. (**E**, **F**) Boxplots of all distal digit cells quantified for 7 individual biological replicates transfected with RCAS-caBMPR1B (**E**) or 5 replicates with RCAS-GFP (**F**).

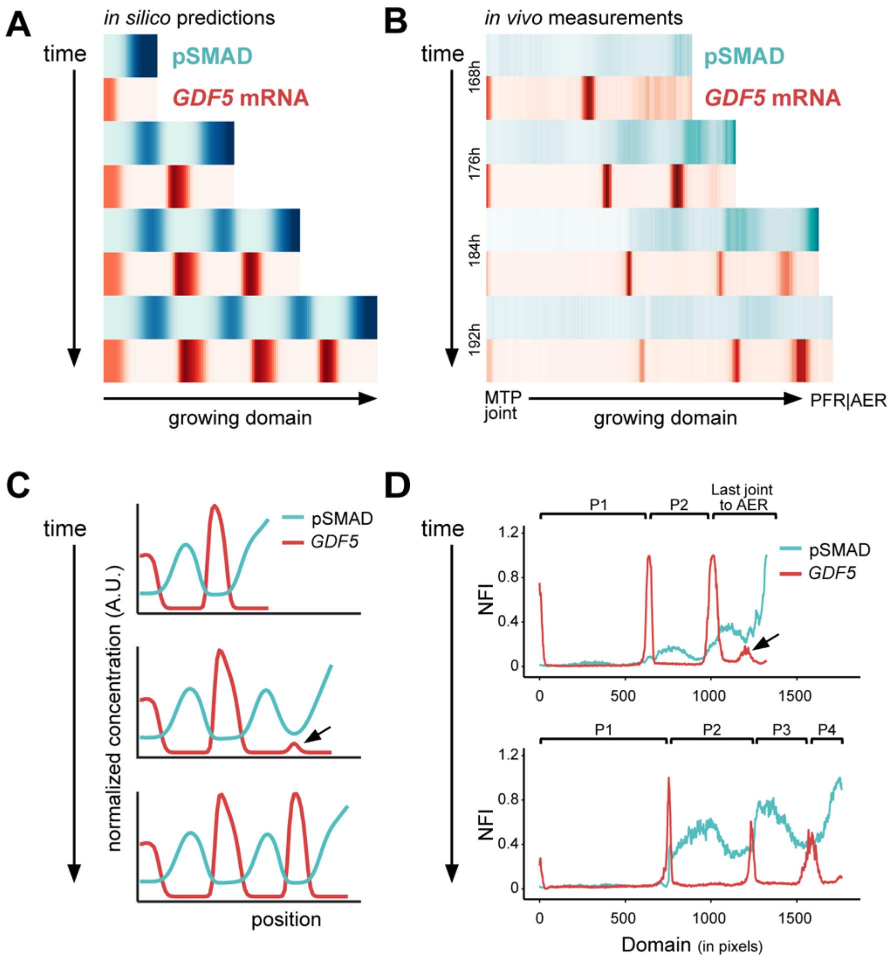

Figure S4. BMP signaling dynamics and patterns during digit development. (**A**) Heat map visualizations of model simulations of normalized pSMAD intensities (top, cyan) and normalized *GDF5* expression (bottom, red), along the proximal-distal axis of a simulated growing digit domain at different timepoints of development. (**B**) Heat map visualization of normalized *in vivo* fluorescence intensities of pSMAD immunohistochemistry (top, cyan) and *GDF5 in situ* hybridizations (bottom, red) along the proximal-distal axis of digit III at different indicated timepoints of development. The very distal zone of high pSMAD corresponds to the PFR. (**C**, **D**) Temporal progression of pSMAD and *GDF5* dynamics *in silico* (**C**) and *in vivo* (**D**). Weak *GDF5* expression initiates in the distal digit where pSMAD is downregulated, marking the future site of the forming joint (see arrows in **C**, **D**).

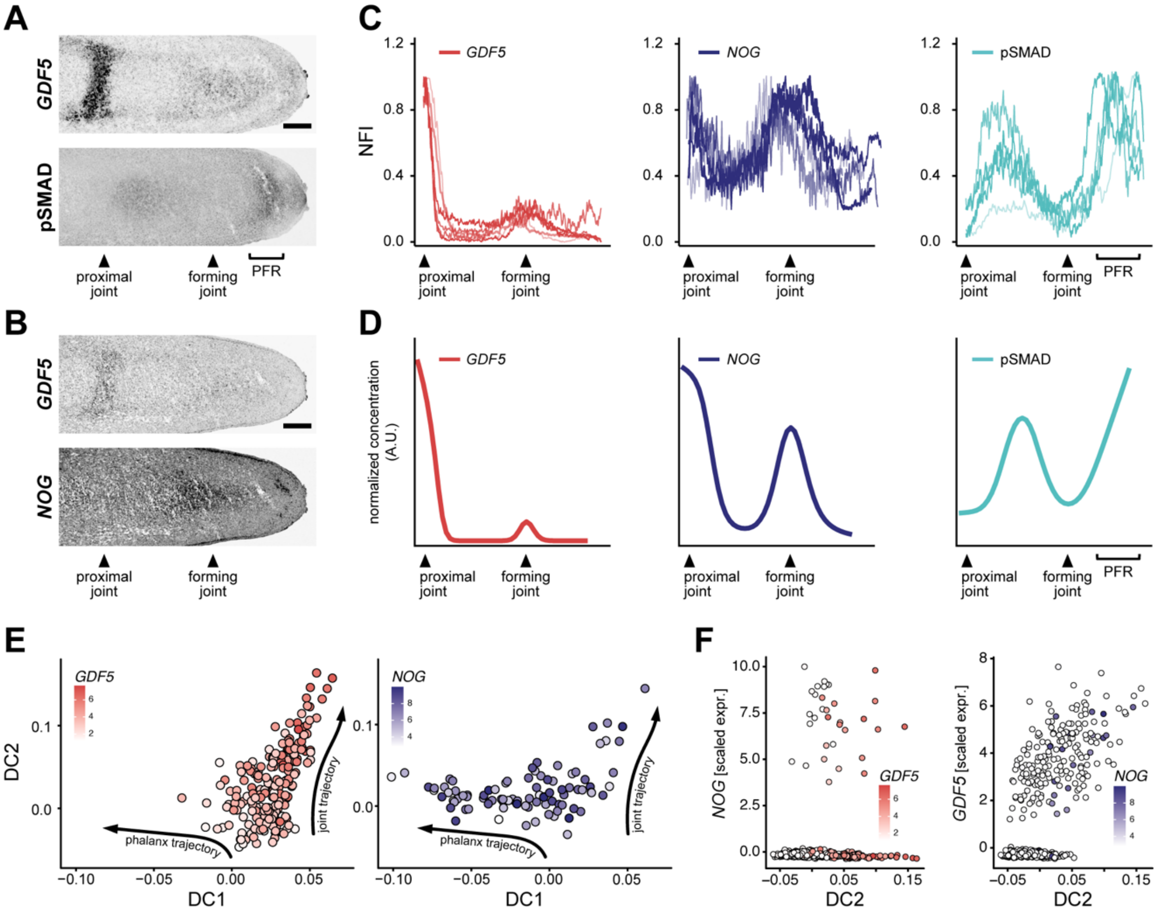

Figure S5. *In vivo* BMP signaling dynamics in newly segmenting phalanges. (**A**, **B**) Neighboring sections of the same digit stained for *GDF5* and pSMAD (**A**) and *GDF5* and *NOG.* Scale bars = 100 µm. (**C**) Superimposition of normalized fluorescence intensity (NFI) curves from multiple embryos for *GDF5* (red), *NOG* (blue), and pSMAD (cyan) in newly forming phalanges. The region from the most distal joint to the digit tip was measured, and individual lengths adjusted to align the corresponding peaks of *GDF5* expression. (**D**) Predicted early *in silico* patterns of *GDF5* (red), *NOG* (blue) and pSMAD (cyan) expression in the distal digit domain. (**E**, **F**) Cellular co-expression of *GDF5* and *NOG* in HH29 scRNA-seq data. (**E**) Cells with a UMI count >0 for either *GDF5* (left) or *NOG* (right) are plotted on the diffusion map, with the scaled expression levels indicated by the respective heatmaps. (**F**) Along the joint trajectory, approximated along DC2, multiple cells show co-expression of *GDF5* and *NOG.*

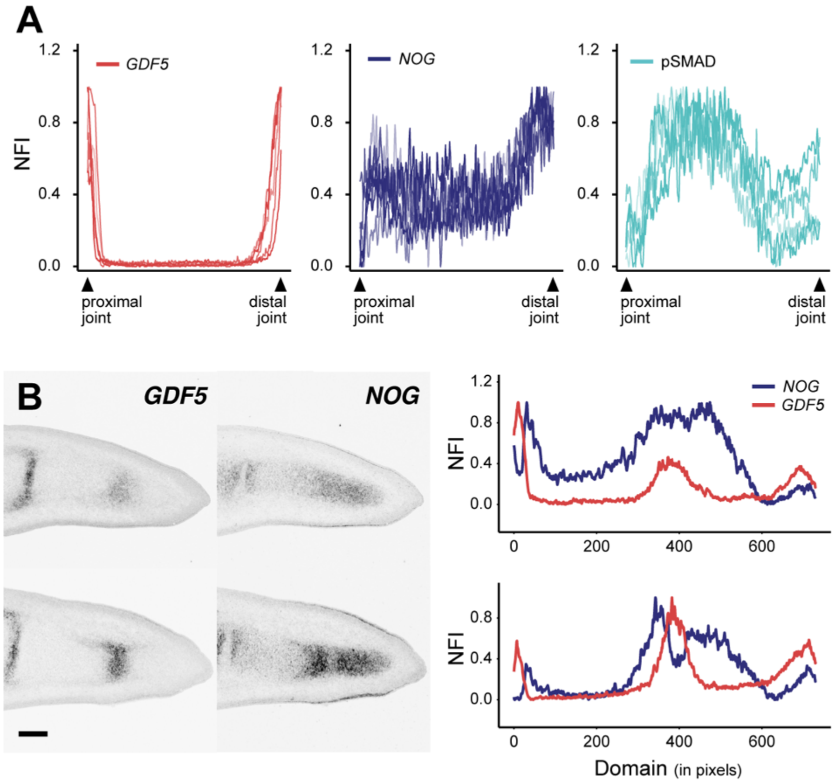

Figure S6. *In vivo* BMP signaling dynamics in maturing phalanges and interphalangeal joints. (**A**) Superimposition of multiple normalized fluorescence intensity (NFI) curves for *GDF5* (red), *NOG* (blue) and pSMAD (cyan) in fully segmented, maturing phalanges. The region spanning two consecutive *GDF5* peaks were measured, and individual lengths were adjusted to align the *GDF5* peaks. (**B**) Spatiotemporal variations in *GDF5*/*NOG* patterns in maturing phalanges. The two images are from embryos collected at the same timepoint, with the lower showing signs of a slightly more developed distal domain. Distal patterns of *NOG* are in phase with *GDF5* expression, but as newly formed phalanges mature and upregulate *GDF5,* the *NOG* domain becomes split into two *GDF5*-adjacent peaks. Scale bar = 100 μm.

A

$$\frac{\partial[\mathrm{GDF5}]}{\partial t} = \frac{\mu_{\mathrm{GDF5}}}{1+([\mathrm{pSMAD}]/K)^{n_1}} - \delta_{\mathrm{GDF5}}[\mathrm{GDF5}] + k_-[\mathrm{COMPLEX}] - k_+[\mathrm{GDF5}][\mathrm{NOG}] + D_{\mathrm{GDF5}}\nabla^2[\mathrm{GDF5}]$$

$$\frac{\partial[\mathrm{NOG}]}{\partial t} = \frac{\mu_{\mathrm{NOG}}}{1+([\mathrm{pSMAD}]/K)^{n_2}} - \delta_{\mathrm{NOG}}[\mathrm{NOG}] + k_-[\mathrm{COMPLEX}] - k_+[\mathrm{GDF5}][\mathrm{NOG}] + D_{\mathrm{NOG}}\nabla^2[\mathrm{NOG}]$$

$$\frac{\partial[\mathrm{COMPLEX}]}{\partial t} = -k_-[\mathrm{COMPLEX}] + k_+[\mathrm{GDF5}][\mathrm{NOG}] + D_{\mathrm{COMPLEX}}\nabla^2[\mathrm{COMPLEX}]$$

$$\frac{\partial[\mathrm{pSMAD}]}{\partial t} = \mu_{\mathrm{pSMAD}}[\mathrm{GDF5}] - \delta_{\mathrm{pSMAD}}[\mathrm{pSMAD}]$$

signaling and transcriptional feedback | degradation | complex formation | diffusion

B

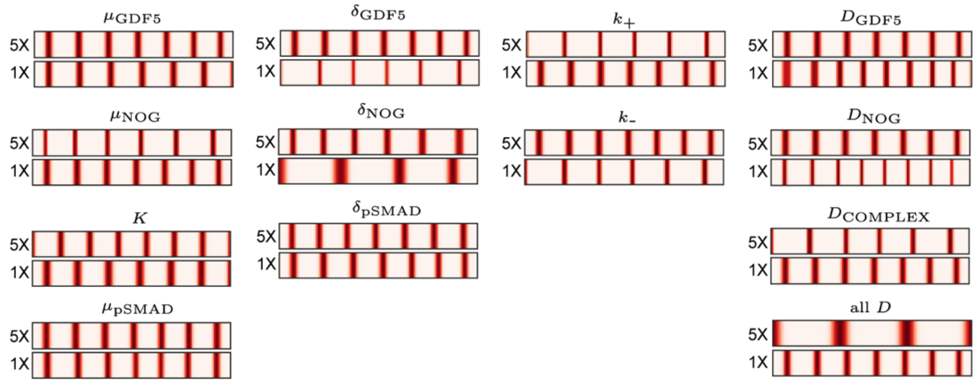

Figure S7. Examples of parameter variations that change pattern wavelength *in silico*. (**A**) Partial differential equations of the BMP-based Turing model, with terms and parameters colored according to the molecular processes they represent (**B**) Model parameters were individually varied across a five-fold range and simulations performed to predict the effect on pattern wavelength. We find that multiple parameters significantly impact pattern wavelength. For the final perturbation (all D, lower right), the diffusion constants for GDF5, NOG and COMPLEX were all varied by the same amount. Parameter definitions: δ_i – degradation rate of protein i; D_i – diffusion constant of protein i; k_+ – complex association rate; k_- – complex dissociation rate; K – the level of pSMAD that results in 50% repression; n_i – Hill coefficients; μ_i – production rate of protein i (for GDF5/NOG this is the maximal production rate; for pSMAD this is the rate per unit concentration of GDF5.

Theory Supplement

S1. Model formulation

We describe the spatiotemporal dynamics of BMP signaling in the digits by the following system of partial differential equations (PDEs):

$$\frac{\partial[\mathrm{GDF5}]}{\partial t} = \mathrm{F_{GDF5}}([\mathrm{pSMAD}]) - \delta_{\mathrm{GDF5}}[\mathrm{GDF5}] + k_-[\mathrm{COMPLEX}] - k_+[\mathrm{GDF5}][\mathrm{NOG}] + D_{\mathrm{GDF5}}\nabla^2[\mathrm{GDF5}]$$
$$\frac{\partial[\mathrm{NOG}]}{\partial t} = \mathrm{F_{NOG}}([\mathrm{pSMAD}]) - \delta_{\mathrm{NOG}}[\mathrm{NOG}] + k_-[\mathrm{COMPLEX}] - k_+[\mathrm{GDF5}][\mathrm{NOG}] + D_{\mathrm{NOG}}\nabla^2[\mathrm{NOG}]$$
$$\frac{\partial[\mathrm{COMPLEX}]}{\partial t} = -k_-[\mathrm{COMPLEX}] + k_+[\mathrm{GDF5}][\mathrm{NOG}] + D_{\mathrm{COMPLEX}}\nabla^2[\mathrm{COMPLEX}]$$
$$\frac{\partial[\mathrm{pSMAD}]}{\partial t} = \mu_{\mathrm{pSMAD}}[\mathrm{GDF5}] - \delta_{\mathrm{pSMAD}}[\mathrm{pSMAD}]$$

Equations 1-4

Here, [...] denotes the concentration of each molecular species, which varies in space within the digit and in time as development proceeds. The processes which define the dynamics of this system are:

<u>*Transcription/secretion:*</u> We assume that GDF5 and NOG are produced and secreted by cells within the digit, at rates $\mathrm{F_{GDF5}}$ and $\mathrm{F_{NOG}}$ respectively. Given that active BMP signaling inhibits *GDF5* transcription, we choose $\mathrm{F_{GDF5}}$ to be a decreasing function of pSMAD. We also consider the possibility of feedback on *NOG* transcription by allowing $\mathrm{F_{NOG}}$ to vary with pSMAD.

<u>*Complex formation:*</u> We assume that extracellular GDF5 and NOG reversibly form a complex: $\mathrm{GDF5} + \mathrm{NOG} \leftrightarrow \mathrm{COMPLEX}$, with an on-rate k_+ and an off-rate k_-. We use mass action kinetics with a 1:1 stoichiometry (1, 2).

<u>*Signaling:*</u> We model BMP signaling *via* the dynamics of pSMAD, assuming that pathway activation increases linearly with the concentration of GDF5 ligand.

<u>*Degradation:*</u> We model the degradation of GDF5 and NOG in the extracellular space with rates δ_{GDF5} and δ_{NOG} respectively, and the intracellular turnover of pSMAD with rate δ_{pSMAD}.

<u>*Diffusion:*</u> We allow all extracellular species (GDF5, NOG, and complex) to diffuse with rates $D_{\mathrm{GDF5}}, D_{\mathrm{NOG}}, D_{\mathrm{COMPLEX}}$ respectively.

Notation: Here we adopt the convention that capitalized variables refer to proteins (e.g., GDF5), whereas italicized variables refer to mRNA gene expression (e.g., *GDF5*).

S2. Deriving necessary conditions for pattern formation

Numerous experimental observations suggest that a Turing instability is responsible for the repetitive patterning of joints within the digit (3). We therefore investigate the conditions that would allow Equations 1-4 to form Turing patterns.

A necessary condition for Turing instabilities is that diffusion causes the homogeneous steady state to become unstable with respect to spatially periodic disturbances. Using well-developed tools for linear instability analysis (4, 5), we begin by computing the steady states of Equations 1-4 in the absence of diffusion. We denote the steady state levels of {GDF5, NOG, complex, pSMAD} as $\{G_0, N_0, C_0, S_0\}$ respectively, and find that:

$$F_{\mathrm{GDF5}}(S_0) = \delta_{\mathrm{GDF5}} G_0$$
$$F_{\mathrm{NOG}}(S_0) = \delta_{\mathrm{NOG}} N_0$$
$$k_- C_0 = k_+ G_0 N_0$$
$$\mu_{\mathrm{pSMAD}} G_0 = \delta_{\mathrm{pSMAD}} S_0$$

Equations 5-8

Next, we explore the response of this homogeneous steady state to small perturbations. We define new variables $\{\Delta g, \Delta n, \Delta c, \Delta s\}$ that describe the normalized deviation of concentrations about the steady state for {GDF5, NOG, complex, pSMAD} respectively (e.g., $\Delta g \equiv ([\mathrm{GDF5}] - \mathrm{G}_0)/\mathrm{G}_0$). Substituting into Equations 1-4, and keeping only the first order (linear) terms, we obtain:

$$\frac{\partial}{\partial \mathrm{t}} \Delta g = \delta_{\mathrm{GDF5}} \mathrm{H}_{GS} \Delta s - \delta_{\mathrm{GDF5}} \Delta g + k_+ N_0 (\Delta c - \Delta n - \Delta g) + D_{\mathrm{GDF5}} \nabla^2 (\Delta g)$$
$$\frac{\partial}{\partial \mathrm{t}} \Delta n = \delta_{\mathrm{NOG}} \mathrm{H}_{NS} \Delta s - \delta_{\mathrm{NOG}} \Delta n + k_+ G_0 (\Delta c - \Delta n - \Delta g) + D_{\mathrm{NOG}} \nabla^2 (\Delta n)$$
$$\frac{\partial}{\partial \mathrm{t}} \Delta c = -k_- (\Delta c - \Delta n - \Delta g) + D_{\mathrm{COMPLEX}} \nabla^2 (\Delta c)$$
$$\frac{\partial}{\partial \mathrm{t}} \Delta s = \delta_{\mathrm{pSMAD}} (\Delta g - \Delta s)$$

Equations 9-12

Here, the H_{ij} terms refer to the normalized sensitivities of the transcriptional functions. Specifically, the term:

$$\mathrm{H}_{GS} \equiv \frac{S_0}{\mathrm{F}_{\mathrm{GDF5}}(S_0)} \frac{\partial F_{GDF5}}{\partial [\mathrm{pSMAD}]} \bigg|_{\mathrm{pSMAD}=S_0}$$

Equation 13

captures how the transcription of *GDF5* depends on the level of pSMAD; we know that $H_{GS} < 0$ since pSMAD inhibits *GDF5*. Similarly,

$$\mathrm{H}_{NS} \equiv \frac{S_0}{\mathrm{F}_{\mathrm{NOG}}(S_0)} \frac{\partial F_{NOG}}{\partial [\mathrm{pSMAD}]} \bigg|_{\mathrm{pSMAD}=S_0}$$

Equation 14

describes the potential feedback between pSMAD activity and *NOG* transcription. If, as published data suggests (6–8), *NOG* is expressed uniformly along the digit then $H_{NS} = 0$. If pSMAD activates *NOG*, then $H_{NS} > 0$; if pSMAD inhibits *NOG*, then $H_{NS} < 0$.

To examine the instability of the steady state to periodic disturbances, we apply the Fourier transform to Equations 9-12, yielding:

$$\frac{\partial}{\partial t}\begin{pmatrix} \Delta g_{\boldsymbol{q}} \\ \Delta n_{\boldsymbol{q}} \\ \Delta c_{\boldsymbol{q}} \\ \Delta s_{\boldsymbol{q}} \end{pmatrix} = -\begin{pmatrix} \delta_{\mathrm{GDF5}} + k_+ N_0 + D_{\mathrm{GDF5}} q^2 & k_+ N_0 & -k_+ N_0 & -\delta_{\mathrm{GDF5}} \mathrm{H}_{GS} \\ k_+ G_0 & \delta_{\mathrm{NOG}} + k_+ G_0 + D_{\mathrm{NOG}} q^2 & -k_+ G_0 & -\delta_{\mathrm{NOG}} \mathrm{H}_{NS} \\ -k_- & -k_- & k_- + D_{\mathrm{COMPLEX}} q^2 & 0 \\ -\delta_{\mathrm{pSMAD}} & 0 & 0 & \delta_{\mathrm{pSMAD}} \end{pmatrix} \begin{pmatrix} \Delta g_{\boldsymbol{q}} \\ \Delta n_{\boldsymbol{q}} \\ \Delta c_{\boldsymbol{q}} \\ \Delta s_{\boldsymbol{q}} \end{pmatrix}$$

Equation 15

We refer to the reaction-diffusion matrix on the right hand side of Equation 15 as $\mathbf{F^{RD}}(q^2)$, mirroring the notation from (4).

A necessary condition for a diffusion-driven instability is that $\det[-\boldsymbol{F^{RD}}(q^2)]$ must become negative for some positive value of q, whilst remaining positive at $q = 0$. Evaluating this determinant gives:

$$\det[-\boldsymbol{F^{RD}}(q^2)] = b_3 q^6 + b_2 q^4 + b_1 q^2 + b_0$$

Equation 16

where the coefficients $\{b_i\}$ may be expressed in terms of the parameters in Equation 15. (The algebraic manipulations are computed using the Symbolic Math Toolbox in MATLAB). Inspecting these coefficients, and recalling that $H_{GS} < 0$, we immediately see that $b_3 > 0$, $b_2 > 0$ and $b_0 > 0$ for all possible model parameters. Using Descartes' rule of signs, we thus find that $b_1 < 0$ is a necessary condition for the polynomial in Equation 16 to change sign for some positive value of q. Explicitly writing the parametric dependence of b_1 gives:

$$(1 - \mathrm{H}_{GS})[D_{\mathrm{COMPLEX}}\delta_{\mathrm{GDF5}}(\delta_{\mathrm{NOG}} + k_+ G_0) + D_{\mathrm{NOG}}\delta_{\mathrm{GDF5}}k_-] + D_{\mathrm{GDF5}}\delta_{\mathrm{NOG}}k_- + D_{\mathrm{COMPLEX}}\delta_{\mathrm{NOG}}k_+ N_0(1 + \mathrm{H}_{NS}) < 0$$

Equation 17

Since we know that $H_{GS} < 0$, this inequality can only be satisfied if the final term is negative, i.e.,

$$\mathrm{H}_{NS} < -1$$

Equation 18

Taken together, it follows that a necessary condition for Equations 1-4 to undergo a Turing instability is that $\mathrm{F_{NOG}}$ is a *decreasing* function of $[\mathrm{pSMAD}]$, i.e., active BMP signaling must inhibit *NOG* transcription. This condition holds regardless of the parameter values chosen in our model, and does not depend on the precise functional form chosen for $\mathrm{F_{GDF5}}$ or $\mathrm{F_{NOG}}$. Together these results place a rather general constraint on the regulatory logic of the system; this is schematized in Figure 2 in the main text.

Extension:

In Equations 1-4, we do not consider the degradation of the complex, since we expect that degradation will be negligible compared to dissociation (i.e., $\delta_{\mathrm{COMPLEX}} \ll k_-$). [Molecular half-lives for extracellular ligands are on the order of hours, e.g., (9), whereas off-rates are typically on the order of minutes, e.g., (10)]. Nonetheless, to relax this assumption, we added complex degradation to Equations 1-4 via a term: $-\delta_{\mathrm{COMPLEX}}[\mathrm{COMPLEX}]$. When we repeated the linear instability analysis, we could derive the same necessary condition for $b_1 < 0$. Inspecting the sign of each term contributing to b_1 using MATLAB, we immediately see that the necessary condition can only be satisfied if $\mathrm{H}_{NS} < 0$, i.e., $\mathrm{F_{NOG}}$ must a *decreasing* function of $[\mathrm{pSMAD}]$. Therefore, our main results (Figure 2) still hold if we consider non-negligible rates for complex degradation, and we confirm via simulation that periodic patterns may form for parameter sets with $\delta_{\mathrm{COMPLEX}} \neq 0$ (Fig.S2E).

S3. Model simulations

Whilst we have derived a necessary condition for pattern formation, we do not have a simplified, analytical expression for a sufficient condition. We therefore turned to simulations to explore whether the system is indeed capable of self-organizing Turing patterns.

We began by simulating Equations 1-4 on a static, one-dimensional domain. Initially we neglected digit growth to focus on the intrinsic, pattern-forming ability of the system, and expected that many qualitative features of the patterns (e.g., phase differences between model variables) would be correctly predicted by simulating on a static geometry.

We chose repressive Hill functions to represent the decreasing functions $\mathrm{F_{GDF5}}$ and $\mathrm{F_{NOG}}$, i.e.,

$$\mathrm{F_{GDF5}} = \mu_{\mathrm{GDF5}}\left(1+\frac{[\mathrm{pSMAD}]^{\mathrm{n_1}}}{\mathrm{K^{n_1}}}\right)^{-1}$$

$$\mathrm{F_{NOG}} = \mu_{\mathrm{NOG}}\left(1+\frac{[\mathrm{pSMAD}]^{\mathrm{n_2}}}{\mathrm{K^{n_2}}}\right)^{-1}$$

Equations 20-21

The resulting PDEs are solved using custom MATLAB scripts which are available at https://github.com/twhiscock/bmp_turing_joint_patterning. We use a pseudo-spectral implicit-explicit numerical method to solve the stiff PDEs. Briefly, we discretize the space into N points, which transforms the PDEs into a system of ODEs. At each time step, we combine the (implicit) backward Euler algorithm with the discrete cosine transform (DCT) to efficiently compute the increments associated with diffusion (and assuming reflective boundary conditions). We then use the (explicit) forward Euler algorithm to compute the increments associated with the reaction terms. We choose appropriate step-sizes (in both time and space) to ensure that the solution is numerically stable; we checked this by halving the time- or spatial step-size in the algorithm and confirmed that we saw no discernible alterations to the predicted patterns. We use homogeneous initial conditions with low amplitude noise added (using normally distributed random numbers) and explore whether the system can then self-organize into periodic patterns.

To visualize the results, we use line plots and heatmaps that describe the predicted concentrations of the extracellular species (GDF5, NOG and complex) as well as the level of BMP signaling (pSMAD). To compare our *in silico* predictions to *in vivo* mRNA measurements, we assume that the term $\mathrm{F_{GDF5}}$ is proportional to *GDF5* mRNA levels, and $\mathrm{F_{NOG}}$ to *NOG* mRNA levels (see Fig 3B).

Incorporating growth dynamics and cell fate commitment

Having investigated the pattern-forming ability of Equations 1-4, we considered how growth dynamics and cell fate commitment would impact the patterning dynamics. We consider a highly simplified scenario to approximate digit growth, following the approach taken in (3). Briefly, we assume that the digit both elongates at its distal edge, as well as uniformly stretches along its length. The combination of distal and uniform growth causes the digit length, L, to increase over time. In addition, we assume that once cells are above a certain distance, L_{pattern}, from the digit tip, their concentrations remain fixed in time (i.e., are no longer governed by Equations 1-4) but can still influence patterning in the distal domain due to diffusion. We emphasize that these assumptions are highly unrealistic and fail to account for the complex processes involved in digit outgrowth, morphogenesis, and cell fate determination. Nonetheless, these crude approximations provide us with proof-of-principle simulations demonstrating how growth might impact predicted expression dynamics.

We considered two types of boundary condition at the growing distal tip, either:

> Reflective-boundary: At each timepoint, we project the concentrations at the digit tip to extend throughout the rest of the domain to enforce reflective boundary conditions (11).

PFR-boundary: Here we assume that there is an additional source of BMP activity at the distal tip of the digit which drives high pSMAD levels at the PFR. Whilst there are several possible ligands that may be relevant *in vivo* (e.g., *Activin* signaling, interdigit BMPs), we make the simplified assumption that a single ligand (termed: $\text{activator}_{\text{pSMAD}}$) is responsible. We assume that the phosphorylation of SMAD is positively regulated by $\text{activator}_{\text{pSMAD}}$, thus modifying Equation 4 to:

$$\frac{\partial[\text{pSMAD}]}{\partial t} = \mu_{p\text{SMAD}_{GDF5}}[\text{GDF5}] + \mu_{\text{pSMAD}_{\text{activator}}}[\text{activator}_{\text{pSMAD}}] - \delta_{pSMAD}[\text{pSMAD}]$$

We assume that $\text{activator}_{\text{pSMAD}}$ is not produced in the patterning region of the digit itself, but rather outside of (i.e., distal to) this domain, from where it can induce a localized increase of pSMAD levels (i.e., the PFR). To account for this, we modify Equations 1-4 to describe alternative dynamics outside of the digit domain:

$$\frac{\partial[\text{activator}_{\text{pSMAD}}]}{\partial t} = \mu_{\text{activator}}^{\text{PFR}} - \delta_{\text{activator}}^{\text{PFR}}[\text{activator}_{\text{pSMAD}}] + D_{\text{activator}}\nabla^2[\text{activator}_{\text{pSMAD}}]$$
$$\frac{\partial[\text{GDF5}]}{\partial t} = -\delta_{\text{GDF5}}^{\text{PFR}}[\text{GDF5}] + D_{\text{GDF5}}\nabla^2[\text{GDF5}]$$
$$\frac{\partial[\text{NOG}]}{\partial t} = -\delta_{\text{NOG}}^{\text{PFR}}[\text{NOG}] + D_{\text{NOG}}\nabla^2[\text{NOG}]$$
$$\frac{\partial[\text{COMPLEX}]}{\partial t} = -\delta_{\text{COMPLEX}}^{\text{PFR}}[\text{COMPLEX}] + D_{\text{COMPLEX}}\nabla^2[\text{COMPLEX}]$$
$$\frac{\partial[\text{pSMAD}]}{\partial t} = \mu_{\text{pSMAD}}^{\text{PFR}} - \delta_{\text{pSMAD}}^{\text{PFR}}[\text{pSMAD}]$$

The form of these equations automatically enforces high pSMAD activity at the distal digit tip, and assumes that GDF5, NOG and COMPLEX are degraded outside of the digit. For simplicity, in the simulations in the main text, we consider the scenario in which $\text{activator}_{\text{pSMAD}}$ has identical properties to GDF5 within the digit (i.e., $\mu_{\text{pSMAD}_{\text{GDF5}}} = \mu_{\text{pSMAD}_{\text{activator}}}, \text{D}_{\text{activator}} = \text{D}_{\text{GDF5}}$ etc.), although we also observed similar qualitative dynamics when we tested several other parameter values associated with $\text{activator}_{\text{pSMAD}}$.

We emphasize that the above equations are designed to generate a distal pSMAD+ domain as simply as possible, and are not meant to accurately capture the specification of the PFR. As discussed in the main text, the regulation of pSMAD levels at the PFR *in vivo* may result from a combination of factors, including: *Activin* signaling at the PFR or interdigital BMPs (12, 13); mechanical interactions at the PFR (14); and regulation of BMPR1B expression by AER-derived FGFs (15). Despite neglecting this complexity in our model, our results still provide proof-of-principle simulations showing how modifications to the distal digit can modulate the patterning dynamics *in silico*.

We found that, regardless of which boundary condition we used, the growth dynamics caused patterning to occur exclusively at the distal tip, with new *GDF5* bands being sequentially added as the digit elongates. These spatiotemporal dynamics were observed for a broad range of parameters, although some parameters (e.g., too low/high values of the growth rate or L_{pattern}) led to patterns that were not observed *in vivo*, such as splitting of phalanges or failure to form repeated patterns, something which is expected from previous studies (3).

Our choice of boundary condition impacted the predicted expression patterns towards the digit tip. For the *reflective-boundary*, we predicted that *GDF5* expression would initiate at the very tip when pSMAD was low, which would then be followed by initiation of pSMAD at the tip and downregulation of *GDF5*. In contrast, for the *PFR-boundary*, we see that pSMAD is always high at the tip (mimicking the PFR), and a nascent *GDF5* band first appears proximal to this region, more closely matching the dynamics that we observe *in vivo* (Figure S4C).

Model parameters

All model parameters used in this manuscript are provided in the accompanying MATLAB scripts (https://github.com/twhiscock/bmp_turing_joint_patterning). Here we will briefly outline the rationale for our parameter choices.

To explore the pattern forming capability of our model, we simulated Equations 1-4 on a static domain and saw that a wide range of parameters led to periodic patterns. We show some example parameter sets in Figure S2C-E, which illustrate how different combinations of diffusivities and degradation rates are compatible with patterning.

For simplicity, for the remaining simulations in the manuscript we focused on a single parameter set, although expect the results to be qualitatively similar for other parameter sets. Figure 3B shows the predicted patterns when simulating with these parameters on a static domain. Figure S7B, also on a static domain, takes the same parameter values as Figure 3B, but varies each one individually over a five-fold range (both lower and higher than the reference). We observe that periodic patterns form reliably across a range of each parameter, but that some parameters significantly affect the wavelength of the resulting pattern.

Using the same parameter set, we performed simulations which also incorporated growth dynamics and cell fate commitment, along with the *PFR-boundary* condition. In Figure S4A&C we predict the sequential addition of new *GDF5* bands at the growing end of the digit. In Figure 4E-G, we mimic experimental perturbations and predict their effect on the *GDF5* pattern (4E: $\mu_{\mathrm{pSMAD}} = 0$; 4F: $\mu_{\mathrm{NOG}} = 0$; 4G: assume extra, localized source of GDF5). In Figure 5B, we explore factors that impact joint number, including variations in growth dynamics ("faster growth": increase growth rate by 1.2X; "longer duration": increase patterning duration by 1.2X) and reaction parameters ("shorter wavelength": increase δ_{NOG} by 2X).

Materials and Methods Supplement

Pseudotime analyses

Pseudotime analysis was performed using a previously published autopod single-cell RNA-sequencing data set (autopods from more than ten ~HH29 embryos were used for tissue dissociation; see (16) for details on quality checks, normalization and cluster identification using *Seurat* v3.1.4 (17)). Three skeletogenic clusters (cls. 3, 15 and 17) were used for further analyses. Using highly variable genes, a diffusion map of the three clusters was calculated with the *R* package *Destiny* (18). Calculation of pseudotime trajectories was performed with *Slingshot* (19). Finally, differential expression analysis along the pseudotime was done using the *MAST* package (20), and expression heatmaps were visualized in *R* studio.

Embryo tissue sampling and processing

Fertilized chicken eggs were incubated at 38°C in a humified incubator and harvested and staged according to the Hamburger-Hamilton developmental table (21). Mouse embryos were isolated and processed for analysis by M. Luxey in accordance with national laws and experimental procedures approved by the Regional Commission on Animal Experimentation and the Cantonal Veterinary Office of the city of Basel (license 1951 to Rolf Zeller and Aimee Zuniga). Embryos were dissected in ice-cold PBS and fixed with 4% paraformaldehyde for 2h on ice. For cryosections, digit tissue was dehydrated in a sucrose gradient up to 30% sucrose/PBS and cryopreserved in OCT (Leica). Cryosectioning was performed at 18 µm thickness.

Immunohistochemistry on cryosections

Cryosections were air-dried for 15 min at room temperature (RT) and washed in Tris-buffered saline (TN: 0.1 M Tris pH 7.5, 0.15 M NaCl). For pSMAD, a step of antigen retrieval was added (1:5000 Proteinase K in TN for 10 min at RT), followed by post-fixation for 5 min in 4% PFA. Endogenous peroxidase was inactivated with 0.3% H_2O_2 in TN for 1h at RT. Slides were blocked in 0.5% BR (Blocking Reagent (Akoya Biociences))/TNT (TN, 0.05% Tween) for 1h at RT and incubated with a primary antibody against pSMAD1,5,9 (Cell Signaling 13820S, rabbit, 1:300) overnight at 4°C. Slides were washed 3 times in TNT and incubate with a biotinylated secondary antibody, followed by streptavidin-conjugated peroxidase incubation and signal amplification using the TSA Plus Cyanine-3 or -5 kits (Akoya Biosciences).

Fluorescent *In situ* hybridization on cryosections

Probe were produced *in vitro* transcription using T3, T7 or SP6 RNA polymerases (Promega) and digoxigenin or fluorescein labeled nucleotides (Roche). *In situ* hybridization was carried out using standard protocols (22), incubating with either anti-digoxigenin or anti-fluorescein-POD antibodies (Roche) diluted at 1:300. Signal amplification was done with 1:50 TSA Plus Cyanine-3 or -5 (Akoya Biosciences) for 1h at RT. For dual-probe RNA *in situ* hybridization, an inactivation step after the first TSA Plus incubation was performed in 0.6% H_2O_2/TNT for 1h at RT. Fluorescent *in situ* hybridization combined with pSMAD staining was performed first, followed by immunohistochemistry in TNT buffer, as described above.

Measurements of normalized fluorescence intensities in developing digits

Fluorescent signals were imaged with a confocal microscope (*Olympus Fluoview FV3000*). Pictures were stitched with the *Pairwise-stitching* plug-in in *Fiji*. After image processing, average fluorescence along the metacarpal to AER axis was measured in *Fiji*, by quantifying pixel intensities

along a line width of 100 pixels (‘Analyze>Plot profile’). Fluorescence intensities were scaled from 0 to 1, based on maximum and minimum values. Finally, plots of normalized fluorescence intensities (NFI) were visualized as line plots or heatmaps in *R* studio.

Mathematical modeling

Full details of the modeling are provided in the theory supplement. Briefly, we constructed a reaction-diffusion model of BMP signaling in the developing digit. We used systems of partial differential equations (PDEs) to describe these dynamics, with molecular interactions as schematized by Fig 2 or Fig 3A. These PDEs were first analyzed using linear instability analysis to derive necessary conditions for pattern formation to occur. We then performed 1D simulations using custom MATLAB scripts. For further details of simulation methods and model parameters, please see *SI Text S3.*

RCAS-caBMPR1B overexpression experiments

Distal digit progenitors were transfected with RCAS viral vectors expressing a constitutively active version of BMPR1B (23) or GFP. Ectopic and mosaic induction of phosphorylated SMAD1/5/9 was verified using IHC against pSMAD and viral *gag* protein (AMV-3C2, DSHB, 1:30). Transfected limbs were processed for *NOG* FISH, combined with IHC against pSMAD or GFP (Abcam, ab13970, 1:1000). We quantified *NOG* and protein marker fluorescence intensities using *CellProfiler* (24). Briefly, we segmented individual cells using DAPI channel-based masks, and therein quantified mean fluorescence intensities for *NOG* mRNA and pSMAD or GFP protein levels. We then ordered the cells based on their mean fluorescence intensities of pSMAD or GFP, and defined cut-off levels to binarize cells into “ON/OFF” (high/low) states using the *R* package *segmented* (25). Mean fluorescence values were scaled within each sample to account for varying intensity distributions across images; this was achieved by computing Z-scores using the *scale* function in R. Effect sizes and confidence intervals were calculated with the *R* package *effsize* (26).

Length measurements of individual digit elements

Chicken hindlimbs were collected along a developmental time series: every 4h, from 128h to 204h of development, and every 8h, from 204h to 336 h. Up to two embryos were collected per time point and both hindlimbs of each embryo were dissected and analyzed. Chromogenic or fluorescent *in situ* hybridization for *GDF5* was performed on early stages, and simple DAPI stains on later stages. Slides were imaged and pictures stitched with the *Pairwise-stitching* plug-in in *Fiji*. Length measurements of individual elements were performed in *Fiji*, by drawing a line from the middle of the proximal joint (as defined by *GDF5* signal, or a gap in the DAPI channel) to the middle of the distal joint, or from the most distal joint to the AER. For total digit length measurements, lengths of all individual elements were summed up. Plots of the digit and phalanx growths were done in *R* studio.

SI References

1. J. Groppe, *et al.*, Structural basis of BMP signalling inhibition by the cystine knot protein Noggin. *Nature* **420**, 636–642 (2002).

2. G. K. Schwaerzer, *et al.*, New insights into the molecular mechanism of multiple synostoses syndrome (SYNS): mutation within the GDF5 knuckle epitope causes noggin-resistance. *J. Bone Miner. Res. Off. J. Am. Soc. Bone Miner. Res.* **27**, 429–442 (2012).

3. J. Cornwall Scoones, T. W. Hiscock, A dot-stripe Turing model of joint patterning in the tetrapod limb. *Development.* (2020) https:/doi.org/10.1242/dev.183699.

4. X. Diego, L. Marcon, P. Müller, J. Sharpe, Key Features of Turing Systems are Determined Purely by Network Topology. *Phys. Rev. X* **8**, 021071 (2018).

5. J. D. Murray, *Mathematical Biology II: Spatial Models and Biomedical Applications*, 3rd edn. Springer (2008).

6. L. J. Brunet, J. A. McMahon, A. P. McMahon, R. M. Harland, Noggin, cartilage morphogenesis, and joint formation in the mammalian skeleton. *Science* **280**, 1455–1457 (1998).

7. C. I. Lorda-Diez, J. A. Montero, J. Rodriguez-Leon, J. A. Garcia-Porrero, J. M. Hurle, Expression and functional study of extracellular BMP antagonists during the morphogenesis of the digits and their associated connective tissues. *PloS One* **8**, e60423 (2013).

8. B.-L. Huang, *et al.*, An interdigit signalling centre instructs coordinate phalanx-joint formation governed by 5'Hoxd-Gli3 antagonism. *Nat. Commun.* **7**, 12903 (2016).

9. A. P. Pomreinke, *et al.*, Dynamics of BMP signaling and distribution during zebrafish dorsal-ventral patterning. *eLife* **6**, e25861.

10. K. Nolan, *et al.*, Structure of Gremlin-2 in Complex with GDF5 Gives Insight into DAN-Family-Mediated BMP Antagonism. *Cell Rep.* **16**, 2077–2086 (2016).

11. C. B. Macdonald, B. Merriman, S. J. Ruuth, Simple computation of reaction-diffusion processes on point clouds. *Proc. Natl. Acad. Sci. U. S. A.* **110**, 9209–9214 (2013).

12. J. A. Montero, *et al.*, Activin/TGFβ and BMP crosstalk determines digit chondrogenesis. *Dev. Biol.* **321**, 343–356 (2008).

13. R. D. Dahn, J. F. Fallon, Interdigital regulation of digit identity and homeotic transformation by modulated BMP signaling. *Science* **289**, 438–441 (2000).

14. C. Parada, *et al.*, Mechanical feedback defines organizing centers to drive digit emergence. *Dev. Cell* **57**, 854-866.e6 (2022).

15. R. Merino, *et al.*, Morphogenesis of Digits in the Avian Limb Is Controlled by FGFs, TGFβs, and Noggin through BMP Signaling. *Dev. Biol.* **200**, 35–45 (1998).

16. C. Feregrino, F. Sacher, O. Parnas, P. Tschopp, A single-cell transcriptomic atlas of the developing chicken limb. *BMC Genomics* **20**, 401 (2019).

17. T. Stuart, *et al.*, Comprehensive Integration of Single-Cell Data. *Cell* **177**, 1888-1902.e21 (2019).

18. P. Angerer, *et al.*, destiny: diffusion maps for large-scale single-cell data in R. *Bioinforma. Oxf. Engl.* **32**, 1241–1243 (2016).

19. K. Street, *et al.*, Slingshot: cell lineage and pseudotime inference for single-cell transcriptomics. *BMC Genomics* **19**, 477 (2018).

20. G. Finak, *et al.*, MAST: a flexible statistical framework for assessing transcriptional changes and characterizing heterogeneity in single-cell RNA sequencing data. *Genome Biol.* **16**, 278 (2015).

21. V. Hamburger, H. L. Hamilton, A series of normal stages in the development of the chick embryo. *J. Morphol.* **88**, 49–92 (1951).

22. E. McGlinn, J. H. Mansfield, Detection of gene expression in mouse embryos and tissue sections. *Methods Mol. Biol*. Clifton NJ 770, 259–292 (2011).

23. H. Zou, R. Wieser, J. Massagué, L. Niswander, Distinct roles of type I bone morphogenetic protein receptors in the formation and differentiation of cartilage. *Genes Dev.* **11**, 2191–2203 (1997).

24. D. R. Stirling, *et al.*, CellProfiler 4: improvements in speed, utility and usability. *BMC Bioinformatics* **22**, 433 (2021).

25. V. M. R. Muggeo, Interval estimation for the breakpoint in segmented regression: a smoothed score-based approach. *Aust. N. Z. J. Stat.* **59**, 311–322 (2017).

26. M. Torchiano, Effsize - a package for efficient effect size computation (2016) https:/doi.org/10.5281/zenodo.196082 (June 21, 2023).
